# An MPER antibody neutralizes HIV-1 using germline features shared among donors

**DOI:** 10.1038/s41467-019-12973-1

**Published:** 2019-11-26

**Authors:** Lei Zhang, Adriana Irimia, Lingling He, Elise Landais, Kimmo Rantalainen, Daniel P. Leaman, Thomas Vollbrecht, Armando Stano, Daniel I. Sands, Arthur S. Kim, IAVI Protocol G Investigators, George Miiro, Jennifer Serwanga, Anton Pozniak, Dale McPhee, Oliver Manigart, Lawrence Mwananyanda, Etienne Karita, André Inwoley, Walter Jaoko, Jack DeHovitz, Linda-Gail Bekker, Punnee Pitisuttithum, Robert Paris, Susan Allen, Pascal Poignard, Dennis R. Burton, Ben Murrell, Andrew B. Ward, Jiang Zhu, Ian A. Wilson, Michael B. Zwick

**Affiliations:** 1 Department of Immunology and Microbiology, The Scripps Research Institute, La Jolla, California 92037 USA; 2 Department of Integrative Structural and Computational Biology, The Scripps Research Institute, La Jolla, California 92037 USA; 3 International AIDS Vaccine Initiative Neutralizing Antibody Center and the Collaboration for AIDS Vaccine Discovery, The Scripps Research Institute, La Jolla, California 92037 USA; 4 Scripps Consortium for HIV/AIDS Vaccine Development, The Scripps Research Institute, La Jolla, California 92037 USA; 5 International AIDS Vaccine Initiative, New York, New York 10004 USA; 6 Department of Medicine, University of California, San Diego, California 92093 USA; 7 Ragon Institute of Massachusetts General Hospital, MIT and Harvard, Cambridge, Massachussetts 02114 USA; 8 Department of Microbiology, Tumor and Cell Biology, Karolinska Institutet, Stockholm, Sweden; 9 Skaggs Institute for Chemical Biology, The Scripps Research Institute, La Jolla, California 92037 USA; 22 Present Address: CTK Biotech, Inc., 3855 Stowe Drive, Poway, California 92064 USA; 23 Present Address: Departments of Medicine, Pathology and Immunology, Washington University School of Medicine, St. Louis, Missouri 63110 USA; 24 Present Address: Institut de Biologie Structurale, Université Grenoble Alpes, Commissariat a l’Energie Atomique, Centre National de Recherche Scientifique and Centre Hospitalier Universitaire Grenoble Alpes, 38044 Grenoble, France; 10 MRC/UVRI Uganda Research Unit on AIDS, Uganda Virus Research Institute, Entebbe, Uganda; 11 St Stephens AIDS Trust, Chelsea and Westminster NHS Foundation Trust, London, UK; 12 NRL, St Vincent’s Institute, Melbourne, Victoria Australia; 13 Zambia Emory HIV Research Project, Lusaka, Zambia and Rwanda-Zambia HIV Research Group, Emory University, Atlanta, GA USA; 14 Projet San Francisco, Kigali, Rwanda and the Rwanda-Zambia HIV Research Group, Emory University, Atlanta, GA USA; 15 CeDReS/CHU Treichville, Abidjan, Côte d’Ivoire; 16 Kenya AIDS Vaccine Initiative, College of Health Sciences, University of Nairobi, Nairobi, Kenya; 17 SUNY Downstate Medical Center, Brooklyn, New York USA; 18 Desmond Tutu HIV Centre, University of Cape Town, Cape Town, South Africa; 19 Faculty of Tropical Medicine, Mahidol University, Bangkok, Thailand; 20 Department of Retrovirology, Armed Forces Research Institute of Medical Sciences, Bangkok, Thailand; 21 Rwanda-Zambia HIV Research Group, Emory University, Atlanta, Georgia USA

**Keywords:** X-ray crystallography, Antibodies, HIV infections, Vaccines

## Abstract

The membrane-proximal external region (MPER) of HIV-1 envelope glycoprotein (Env) can be targeted by neutralizing antibodies of exceptional breadth. MPER antibodies usually have long, hydrophobic CDRH3s, lack activity as inferred germline precursors, are often from the minor IgG3 subclass, and some are polyreactive, such as 4E10. Here we describe an MPER broadly neutralizing antibody from the major IgG1 subclass, PGZL1, which shares germline V/D-region genes with 4E10, has a shorter CDRH3, and is less polyreactive. A recombinant sublineage variant pan-neutralizes a 130-isolate panel at 1.4 μg/ml (IC_50_). Notably, a germline revertant with mature CDR3s neutralizes 12% of viruses and still binds MPER after DJ reversion. Crystal structures of lipid-bound PGZL1 variants and cryo-EM reconstruction of an Env-PGZL1 complex reveal how these antibodies recognize MPER and viral membrane. Discovery of common genetic and structural elements among MPER antibodies from different patients suggests that such antibodies could be elicited using carefully designed immunogens.

## Abstract (web summary)

Here, the authors identify a broadly neutralizing antibody from an HIV-infected person that recognizes the membrane-proximal external region (MPER) of HIV envelope glycoprotein (Env) and has a short CDRH3 and low polyreactivity. Structural analysis shows how the antibody binds the MPER and Env on the viral membrane.

## Introduction

A key goal in HIV vaccine design is to elicit broadly neutralizing antibodies (bnAbs)^1^. Most bnAbs to HIV-1 have been cloned from elite donors whose plasma shows broad neutralizing activity. These bnAbs target six distinct sites on the HIV-1 envelope glycoprotein (Env) spike, including the CD4-binding site (CD4bs), V2 apex, N332/V3 base supersite, silent face, gp120-gp41 interface (including fusion peptide), and membrane-proximal external region (MPER). As bnAbs arise from complex affinity maturation pathways, efforts are underway to dissect the structural and genetic bases of bnAb function to uncover common elements that can simplify vaccine design^2^.

MPER bnAbs show outstanding breadth, neutralizing up to >98% primary isolates, but have uncommon features^3^. MPER bnAbs are often from the IgG3 subtype, which has caused speculation that eliciting these bnAbs involves certain B-cell subsets, class-switching, or a specific hinge region^4,5^. Although 2F5, 4E10, 10E8, DH511, DH517, VRC46, and VRC43.01 are IgG3s, the MPER bnAb VRC42 was isolated as an IgG1 from the same subject as the latter two bnAbs^5^. Notably, long heavy complementarity-determining region (CDR) H3 loops with aromatic residues at the tip facilitate bnAb binding to the hydrophobic MPER and nearby membrane^6^. However, B-cell receptors (BCRs) with long and hydrophobic CDRH3s tend to be downregulated during B-cell ontogeny^7^ and some MPER bnAbs, e.g., 4E10, are mildly polyreactive^8^. Further, 4E10 knock-in mice exhibit B-cell tolerance via clonal deletion and anergy^9^. BnAbs 10E8 and DH511 were recently shown to recognize a similar epitope as 4E10 with less polyreactivity and higher potency^10,11^, but key information is missing on the precise antigens and mechanisms that drove their evolution.

The hydrophobic MPER is often truncated from Env constructs to render soluble gp140 trimers^12^; thus, the MPER has been commonly studied in isolation. MPER peptide, N_671_WFDITNWLWYIK_683_, adopts a mainly α-helical conformation with W_672_–D_674_ in a 3_10_ helix when bound to 4E10 and is fully helical when bound to 10E8 and DH511^11,13^. However, MPER peptides constrained as an α-helix have not elicited nAbs^3^. One issue is that the membrane can hinder antibody access to the MPER on the virus^6^. Further, cryogenic electron microscopy (cryo-EM) reconstructions have revealed interaction of 10E8 with N-linked glycans on membrane-extracted Env at positions 88 and 625^14^. Thus, elicited antibodies should accommodate membrane and adjacent glycans on the Env trimer. MPER accessibility increases transiently when Env binds to CD4 receptor, just prior to co-receptor binding and virus entry into host cells, but structural details of this transient state are lacking.

Recently, vaccine design has focused on targeting common elements among certain bnAb precursors. For example, VRC01-class CD4bs antibodies typically use germline gene *V*_*H*_*1-2*, for which specific immunogens have been designed^15^. Germline-encoded residues important for Env recognition by different V2 apex bnAbs have also been identified^16^, whereas other bnAb precursors recognize a transmitted-founder (T/F) Env^17^. Germline revertants of many bnAbs do not bind to Env, although some somatic hypermutations (SHM) are dispensable with 4E10 and 10E8^18,19^. Interestingly, a recently described 4E10-like bnAb, VRC42, plus two other MPER bnAb lineages with limited SHM, were elicited by a single T/F Env^5^.

Here we report on MPER bnAb PGZL1 and its lineage variants, which, along with 4E10 and VRC42, share the usage of *V*_*H*_*1-69* and *V*_*K*_*3-20* V-region germline genes, as well as D-region allele D3-10. PGZL1 is an IgG1 that has a relatively short CDRH3 with limited hydrophobicity and is less polyreactive than 4E10; PGZL1 germline revertants can still bind Env and neutralize HIV. Thus, 4E10-like antibodies may be more probable to elicit in humans than previously thought and, therefore, provide clues for vaccine design.

## Results

### Identification of a 4E10-like antibody from an African donor

**Fig. 1:**
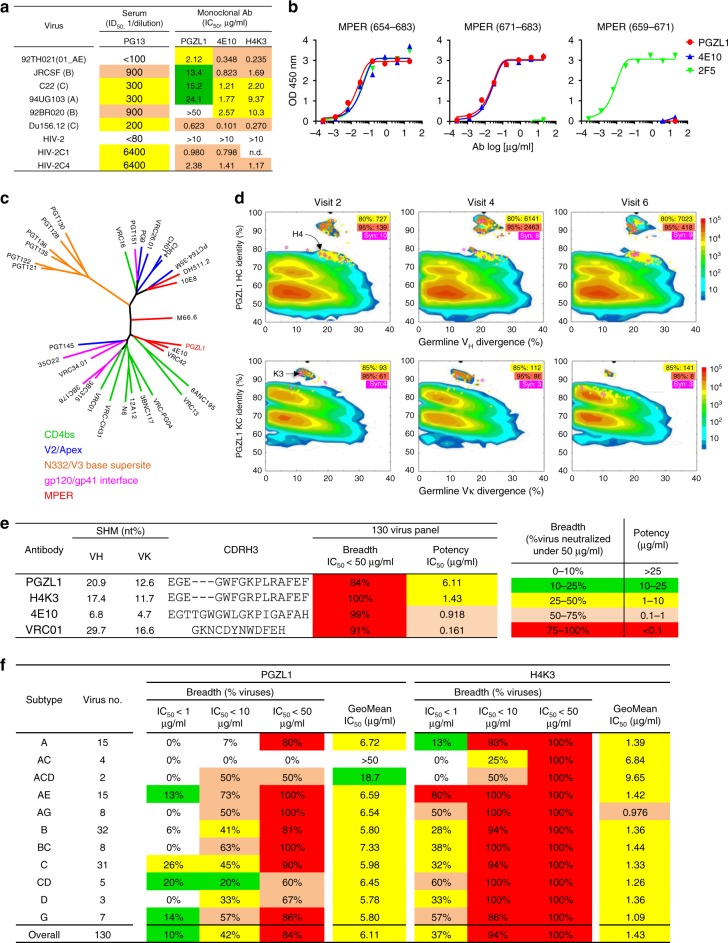
Properties of an MPER-targeted bnAb**. a** Neutralization of HIV-1 six-virus panel and HIV-2 (HIV-1 MPER) chimeras by PG13 plasma and monoclonal antibodies PGZL1, 4E10, and H4K3. **b** ELISA binding of PGZL1 to MPER peptides, using 4E10 and 2F5 as controls. **c** Maximum likelihood (ML) tree of HC variable regions of described bnAbs, colored by Env specificity. **d** Divergence/identity analysis of donor PG13 antibody repertoire over three visits in 9 months. NGS-derived antibody chains are plotted as a function of sequence identity to PGZL1 and divergence from their putative germline genes. Colors indicate sequence density. Sequences with a CDR3 identity of ≥80/85% (HC/KC) and with a CDR3 identity of ≥95% are shown as yellow and orange dots on the 2D plots, with the number of sequences highlighted in yellow and orange shades, respectively. Sequences bioinformatically selected for synthesis are shown as magenta stars on the 2D plots, with the number of sequences (Syn) highlighted in magenta shade. **e**, **f** Neutralization breadth and potency of PGZL1 and H4K3 against a 130-virus panel (**e**) and the same data as in **e** but subdivided by HIV subtype (**f**).

Plasma from visit 2 (out of a total of six visits) of an HIV-1-infected South African donor, PG13, in the IAVI Protocol G cohort^20^ was tested for neutralization against HIV-2 (HIV-1 MPER) chimeric viruses. The titer was 1:6400 (ID_50_) against 4E10-sensitive chimeras C1 and C4, but the 2F5-sensitive C3 chimera was not neutralized, suggesting a 4E10-like antibody (Fig. 1a and Supplementary Table 1). The plasma also neutralized five of a six-virus panel (Fig. 1a). MPER peptide partially blocked plasma neutralization of primary isolate Du156.12 and the C1 chimera (Supplementary Table 1). MPER-positive B cells from PG13 visit 5 were then sorted by fluorescence-activated cell sorting (FACS; see Methods), and the resulting heavy-chain (HC) and light-chain (LC) variable regions were cloned into IgG vectors. Antibody PGZL1 bound to full-length MPER and a 4E10-specific peptide, but not to a 2F5-specific peptide (Fig. 1b).

DNA sequencing revealed PGZL1 was from subclass IgG1, whereas most MPER bnAbs are IgG3s (Supplementary Table 2). Phylogenetic analysis showed sequence homology between PGZL1, VRC42.01, and 4E10, due to shared germline genes *V*_*H*_*1-69*, *V*_*K*_*3-20*, and *D*_*H*_3-10*01 (Fig. 1c and Supplementary Table 2). PGZL1 has a high degree of SHM (20.9% nucleotides for HC and 12.6% for LC; Fig. 1e), and five HC and two LC mutations match those in 4E10 (Supplementary Fig. 1). PGZL1, VRC42.01, and 4E10 use different J-genes. The PGZL1 CDRH3 contains 15 residues, 3 residues shorter than 4E10, and equal in length to VRC42.01. The three CDRH3s share some amino acids not only from the same D-gene, e.g., W_99_, G_100a_, but also at positions E_95_, G_96_, G_98_, K_100b_, P_100c_, and A_100f_, which may have arisen from N-additions or SHM **(**Supplementary Fig. 1).

### Broad HIV neutralization by PGZL1 lineage antibodies

We tested PGZL1 against a six-virus panel and found that it neutralized fiveviruses with an IC_50_ of 5.8 μg/ml, which was sixfold less potent than 4E10 (Fig. 1a). To identify more potent PGZL1 variants, we analyzed BCR transcripts of donor PG13 B cells taken from three visits (visit 2, 4, and 6) in a short 9-month span using a next-generation sequencing (NGS) pipeline^21^ (Supplementary Table 3). The PG13 repertoire profiles were similar among visits, with PGZL1 germline gene *V*_*H*_*1-69* accounting for 3.9–7.2% of the repertoire, whereas *V*_*H*_*4-34* and *V*_*H*_*4-59* composed up to 13% (Supplementary Fig. 2). Two-dimensional identity/divergence analysis (Fig. 1d) and CDRH3-based lineage tracing showed two groups of PGZL1-related HCs: one group formed an island with full-length identity of 85–100% and the other was created by a long stretch of up to 80% identity, suggesting a sublineage related to PGZL1. As earlier PG13 peripheral blood mononuclear cells (PBMCs) and plasma samples were depleted or unavailable, the time of lineage division could not be determined. Overall, the PGZL1 lineage appears to be highly evolved, showing similar patterns to the VRC01-class bnAbs that often diverge into many sub-lineages^22^.

For functional studies, a clustering analysis of CDRH3s with at least 80% identity allowed a broad selection of 27 HCs from the PGZL1 sub-lineages (Supplementary Fig. 3a and Supplementary Table 4). CDRH3 remained conserved in length and sequence, perhaps due to affinity maturation against the conserved MPER. We paired the HCs with the PGZL1 LC and tested these antibodies against Du156.12. The best neutralizers, H4 and H8 from Visit 2 (Supplementary Table 4), had two- to fourfold lower IC_50_s than PGZL1 (Supplementary Fig. 3b). Of note, H4 and H8 seemed to come from a distinct sublineage having low sequence identity (≤83%) with respect to PGZL1 HC (Fig. 1d). H4 and H8 HCs were then paired with 10 similarly chosen NGS variant LCs (Supplementary Table 4). PGZL1 antibodies, H4K3 and H8K3, which contain the LC K3 from donor Visit 2, neutralized Du156.12 and 92TH021 with a further two- to fourfold decrease in IC_50_s (Supplementary Fig. 3c). PGZL1.H4K3 (hereafter H4K3) was chosen for more detailed characterization. H4K3 has less SHM than PGZL1, i.e., 17.4%/11.7% vs. 20.9%/12.6% at the nucleotide level for HC/LC, respectively, consistent with H4K3 being sampled 5 months prior to PGZL1.

We used a larger panel of HIV-1 primary isolates to examine breadth and potency of PGZL1 and H4K3. Notably, H4K3 neutralized 100% viruses at ≤ 50 μg/ml (*n* = 130), with an IC_50_ of 1.43 μg/ml (Fig. 1e, f and Supplementary Table 5). 4E10 gave similar results, neutralizing 99% isolates with an IC_50_ of 0.92 μg/ml. PGZL1 neutralized 84% viruses, with an IC_50_ of 6.11 μg/ml. The IC_50_s of PGZL1 and H4K3 are tightly correlated (*r* = 0.82, *p* < 0.0001), suggesting similar neutralization mechanisms (Supplementary Fig. 3d). VRC01 neutralized 91% viruses with an IC_50_ of 0.161 μg/ml. Overall, H4K3 is exceptionally broad and equipotent with 4E10, which has been reported to protect against SHIV challenge in monkeys^23^.

### PGZL1 germline revertant binds the MPER and neutralizes HIV

**Fig. 2:**
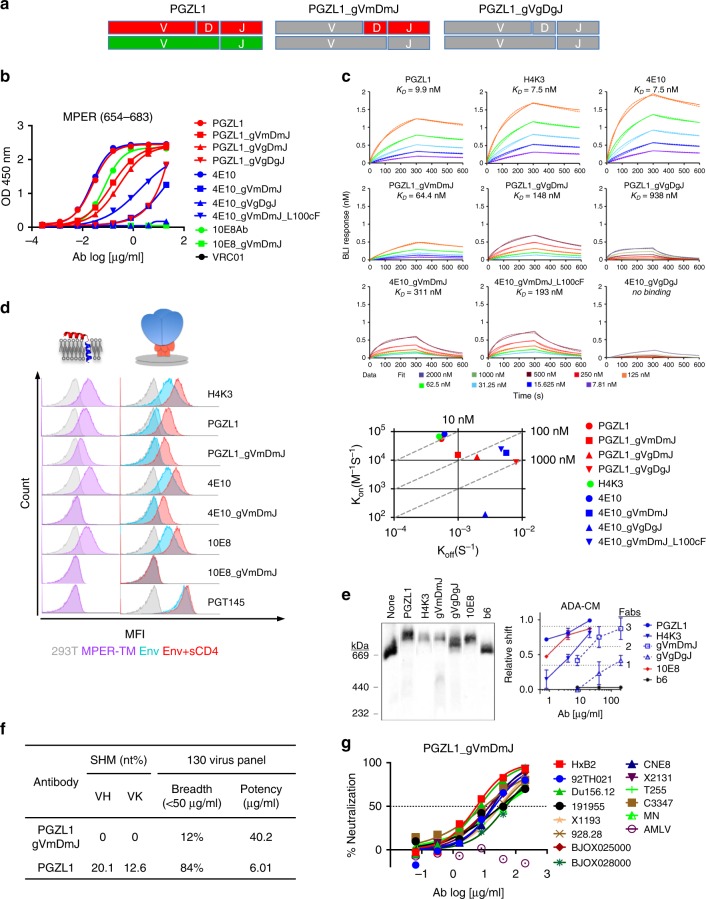
Characterization of germline-reverted antibody PGZL1 gVmDmJ. **a** Cartoon of mature PGZL1 V_H_ (red; top) and Vκ (green; bottom) subdivided by V, D, and J regions, and germline reversions (gray) to create PGZL1 gVmDmJ (middle) and PGZL1 gVgDgJ (right). **b** ELISA binding of PGZL1 germline revertants to MPER peptide, using analogous 10E8 and 4E10 controls. **c** BLI-binding kinetics of PGZL1 variants to immobilized MPER peptide (top panels). 4E10 variants were also used for comparison. *k*_on_ and *k*_off_ of antibodies are shown on a scatter plot with affinity constant, *K*_D_, as dashed lines (bottom). **d** Cells expressing MPER-TM (purple histograms, left) were stained in flow cytometry by mature and germline-reverted antibodies at 2 μg/ml. HIV Env (right) in the presence and absence of soluble CD4 (sCD4; red and blue histograms, respectively) were stained by mature and germline-reverted antibodies at 2 and 10 μg/ml, respectively. **e** BN-PAGE Env mobility shift assay. HIV-1 virions were incubated with Fab PGZL1 and H4K3 (20 µg/ml), or PGZL1 gVmDmJ and gVgDgJ (200 µg/ml). 10E8 (20 µg/ml) and non-neutralizing antibody b6 (200 µg/ml) were used as positive and negative controls, respectively. Relative shift and stoichiometry of Fab to Env was quantified. The error bars represent the SD of *n* = 2 biologically independent experiments. **f** Neutralization potency and breadth of PGZL1 gVmDmJ against a 130-virus panel of HIV-1 in TZM-bl assay at 200 μg/ml. **g** Neutralization of 13 isolates sensitive to PGZL1 gVmDmJ chosen from the 130-virus panel. Source data for **b**–**g** are provided as a Source Data file.

Germline revertants of 4E10^24^ and 10E8^19^ reportedly do not neutralize HIV-1. We reverted the V-region of PGZL1 to the most homologous germline alleles, i.e., *V*_*H*_1-69*06, *V*_*K*_3-20*01, while leaving the CDR3s unchanged, thus creating PGZL1 gVmDmJ; in a second antibody PGZL1 gVgDgJ, putative reversions were also made to *D*_*H*_3-10*01 and *J*_*H*_3*01 (Fig. 2a and Supplementary Fig. 4a). Surprisingly, PGZL1 gVmDmJ and gVgDmJ still bound the MPER peptide, but with a 6- to 12-fold increase in EC_50_ (Fig. 2b); DJ-reverted PGZL1 gVgDgJ also bound, albeit with a 369-fold increase in EC_50_. Analogous revertants 4E10 gVmDmJ, 4E10 gVgDgJ, and 10E8 gVmDmJ showed little or no such binding as previously reported (Fig. 2b)^18,19^.

Using biolayer interferometry (BLI), PGZL1, PGZL1_gVmDmJ, and PGZL1_gVgDgJ bound with affinities (*K*_D_) ranging from 9.9 nM to 64.4 nM and 938 nM, respectively, mostly due to different off-rates (Fig. 2c). We created another mutant, PGZL1 gVgDmJ, to test the effect of changes in D_H_. PGZL1 gVgDmJ bound the MPER peptide with a *K*_D_ of 148 nM, indicating that reversions in both D_H_ and J_H_ affect the PGZL1 off-rate. PGZL1 and H4K3 had similar *K*_D_s of 9.9 nM and 7.5 nM, respectively, but on- and off-rates of H4K3 to MPER peptide were faster by about 24 and 8%, respectively (Fig. 2c).

To test whether the gVmDmJ germline revertants could bind to the MPER on the cell surface, we created a 293T cell line that displays the MPER and transmembrane domain (MPER-TM_654–709_). The PGZL1 gVmDmJ revertant, but not 4E10 or 10E8 revertants, also stained MPER-TM cells, albeit not as well as mature PGZL1, H4K3, 4E10, and 10E8 (Fig. 2d). We then assessed antibody staining of a cell line that overexpresses Env^25^. We observed moderate staining of high-Env cells by PGZL1 in the absence of sCD4 and strong staining by 4E10, 10E8, and H4K3 (Fig. 2d). Cell surface Env was not stained by the revertants at a concentration of 10 μg/ml (Fig. 2d). Addition of sCD4 enhanced MPER bnAb staining of Env cells, as expected; notably, sCD4 also enabled staining by PGZL1 gVmDmJ and 4E10 gVmDmJ, but not by 10E8 gVmDmJ. Of note, the 4E10 revertant reportedly bound weakly to a deglycosylated MPER-containing gp140^26^.

We also assessed binding of PGZL1 variants to the detergent-extracted Env ADA-CM, which is the ADA Env containing trimer stabilizing mutations ΔN139, ΔI140, N142S, I535M, L543Q, K574R, H625N, T626M, and S649A^27^. In a blue-native polyacrylamide gel electrophoresis (BN-PAGE) mobility shift assay, both PGZL1 and H4K3 shifted Env (Fig. 2e). Notably, PGZL1 gVmDmJ and gVgDgJ also shifted Env, but gVgDgJ required a higher concentration and did not reach a binding stoichiometry of three Fabs per trimer. BnAb 10E8 and non-nAb b6 controls showed the predicted gel shift and lack of shift, respectively (Fig. 2e). Similar results were observed using Envs HxB2 and Du156.12 (Supplementary Fig. 4b). Hence, PGZL1 gVgDgJ binds to Env when detergent-extracted from membrane, but not on the cell surface, whereas PGZL1 gVmDmJ binds both solubilized and cell surface Env.

Strikingly, PGZL1 gVmDmJ neutralized 12% of isolates at 50 μg/ml and 28% of viruses at 200 μg/ml in a dose-dependent manner (Fig. 2f, g and Supplementary Table 5). The IC_50_s of PGZL1 and PGZL1 gVmDmJ correlated modestly (*r* = 0.52, *p* = 0.0013) (Supplementary Fig. 3d). We tested viruses especially sensitive to PGZL1 gVmDmJ against additional revertants of PGZL1 and 4E10 (Supplementary Fig. 4). No virus was neutralized by PGZL1 gVgDgJ or 4E10 gVgDgJ at 200 μg/ml (Supplementary Fig. 4c), despite the ability of PGZL1 gVgDgJ, but not 4E10 gVgDgJ, to bind the MPER (Fig. 2b). However, 4E10 gVmDmJ neutralized 92TH021 and CNE8, whereas 4E10 gVmDmJ_L_100c_F, which has the *D*_*H*_3-10*01-encoded F_100c_ and binds MPER ~ 2-fold better than 4E10 gVmDmJ (Fig. 2c), neutralized these isolates more potently in addition to T255 and X1193 (Supplementary Fig. 4c). Thus, ~ 1.4% isolates were neutralized by revertants of PGZL1 and 4E10, which had the D-gene-encoded F_100_ (L_100c_ in 4E10; Supplementary Fig. 1a) in the context of mature CDR3s.

4E10 neutralizes HIV-1 by a slow and/or post-CD4-attachment mechanism, as it can be washed off the virions prior to adding to target cells^10^. We performed washouts with PGZL1 gVmDmJ, using VRC01 as a control that binds directly to Env and resists washout. Neutralization of Du156.12 by PGZL1 gVmDmJ was partially lost on washout but was unchanged with VRC01 (Supplementary Table 6). Notably, PGZL1 gVmDmJ neutralized 928.28, BJOX025000, HxB2, and 92TH021, albeit modestly, following the washout (Supplementary Table 6). Thus, PGZL1 gVmDmJ binds weakly to and neutralizes some primary isolates prior to receptor engagement.

### PGZL1 uses 4E10-like features but has limited polyreactivity

**Fig. 3:**
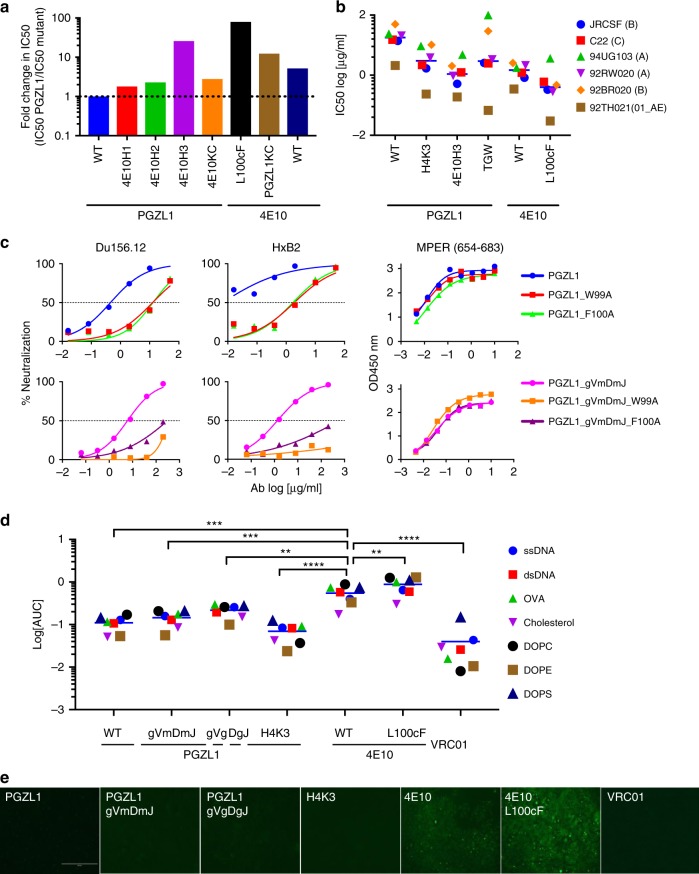
Dominant role of CDRH3 in PGZL1 HIV-1 neutralization by D-gene-encoded residues. **a** Fold decrease in neutralization (IC_50_) of isolate 92TH021 relative to wild-type PGZL1 by CDR grafts and LC substitution from 4E10 (left), and by 4E10 substitutions L_100c_F and PGZL1 LC (right). **b** Neutralization (log IC_50_) of a six-virus panel by PGZL1 and 4E10 variant antibodies. **c** Effect of Ala substitutions in *D*_*H*_-encoded residues W99 and F100 on the ability of PGZL1 mature (top panels) and inferred germline antibodies (bottom panels) to neutralize Du156.12 and HxB2 (left panels), as well as to bind MPER peptide in an ELISA (right panels). **d** Antibody polyreactivity in an ELISA as a function of area under the curve (AUC) of PGZL1 and 4E10 variant antibodies against nonspecific antigens. VRC01 is a negative control. Two-way ANOVA multiple comparisons was used to compare the difference between groups (*n* = 7, **p = 0.0062, ***p = 0.0001, ****p < 0.0001). **e** Immunofluorescence staining of HEp-2 cells. Antibodies were tested at 50 μg/ml using 4E10 and VRC01 as positive and negative controls, respectively; images are at ×200 magnification and the scale bar is 400 µm. Source data for **b**–**d** are provided as a Source Data file.

To dissect PGZL1 activity, we created chimeras of PGZL1 and 4E10, and tested their ability to neutralize 92TH021. Engraftment of 4E10 CDRs H1 and H2 onto PGZL1 produced a modest ~ 2-fold increase in potency **(**Fig. 3a). Engrafting CDRH3 of 4E10 onto PGZL1 caused a 20-fold increase in neutralization. Substituting the LC or HC of PGZL1 with that of 4E10 produced 3-fold and 10-fold increases in potency, respectively. Thus, PGZL1 becomes more potent using 4E10 LC or HC CDRs, and in particular its CDRH3.

The most prominent difference in CDRH3s of PGZL1 and 4E10 is the additional Thr-Gly-Trp (TGW) motif in 4E10 (Supplementary Fig. 1a). This motif increases CDRH3 hydrophobicity (Supplementary Fig. 1b); thus, this might enhance neutralization via membrane interaction. Of note, a putative variant of VRC42.01, termed VRC42.N1, was identified using plasma proteomics and contains a Glu-Gly-Trp (EGW) insertion at the analogous position in VRC42^5^. Indeed, insertion of TGW improved PGZL1 neutralization roughly threefold, but the fold-change varied among isolates in the six-virus panel, indicating paratope context also affected neutralization. CDRH3s of PGZL1 and 4E10 differ at position 100, which is the *D*_*H*_3-10*01-encoded F_100_ in PGZL1, but somatically mutated to L_100c_ in 4E10. The hydrophobic mutant 4E10 L_100c_F neutralized threefold more potently than wild-type 4E10 (Fig. 3b) and was the most potent 4E10 mutant of those we tested or could find in the literature.

As PGZL1 and 4E10 share the *D*_*H*_3-10*01 germline gene (Supplementary Fig. 4a), we asked whether Ala mutants of *D*_*H*_3-10*01-encoded W_99_ and F_100_ affect the activity of PGZL1 and PGZL1 gVmDmJ. Mutations W_99_A and F_100_A decreased neutralization of Du156.12 and HxB2 by these antibodies more than tenfold (Fig. 3c). Notably, W_99_A and F_100_A did not affect binding of either antibody to MPER peptide in enzyme-linked immunosorbent assay (ELISA) (Fig. 3c), suggesting their activity may relate more to MPER recognition on virions. Hence, *D*_*H*_3-10*01-encoded W_99_ and F_100_ are crucial for neutralization by PGZL1.

BnAb 4E10 exhibits polyreactivity^8^, which is of interest, as 4E10 knock-in mice show B-cell tolerance control^9,24^. By ELISA, we verified that 4E10 was polyreactive towards single-stranded DNA, double-stranded DNA (dsDNA), ovalbumin (Ova), and lipids, cholesterol, 1,2-dioleoyl-sn-glycero-3-phosphocholine (DOPC), 1,2-dioleoyl-sn-glycero-3-phosphoethanolamine (DOPE), and 1,2-dioleoyl-sn-glycero-3-phospho-L-serine (DOPS), whereas a negative control bnAb VRC01 showed no such binding (Fig. 3d). Of note, 4E10 L_100c_F showed higher binding than 4E10 to the above panel. PGZL1, gVmDmJ, gVgDgJ, and H4K3, all showed less nonspecific binding than 4E10 to the same antigens. Similarly, HEp-2 cells were stained by 4E10, whereas PGZL1, gVmDmJ, gVgDgJ, and H4K3, all showed less staining (Fig. 3e).

### PGZL1 structure closely resembles 4E10

**Fig. 4:**
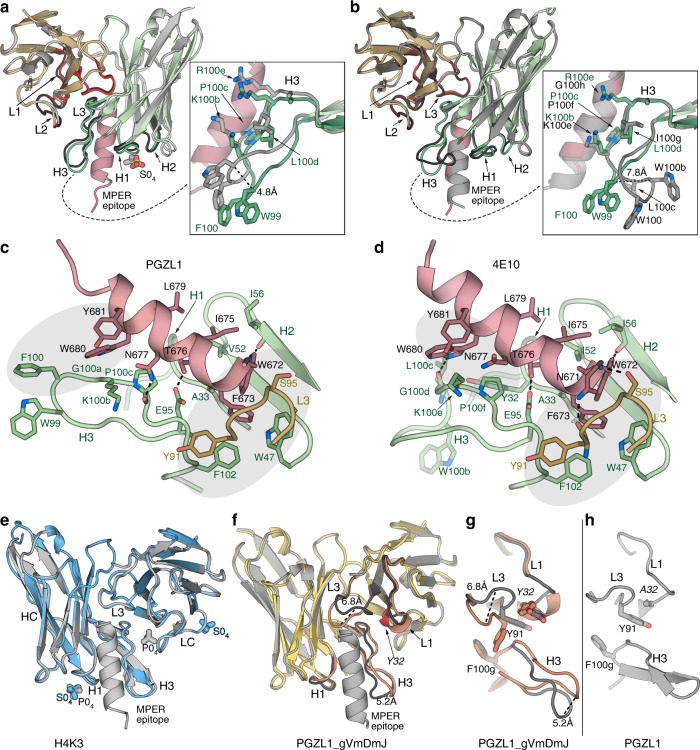
PGZL1 variant crystal structures and comparison with 4E10. **a** Superposition of the crystal structures of the mature PGZL1 variable domain (from the Fab) bound to MPER_671-683_ (wheat, LC; green, HC; pink, MPER) and unbound PGZL1 (gray). CDRs of the bound structure are shown in red (LC) and green (HC), and CDRs of the unbound structure are shown in black. Inset: superposition of free and bound CDRH3 with residues near the MPER shown as sticks. **b** Superposition of PGZL1–MPER_671-683_ and 4E10-MPER_671-683_ (gray; PDB 2FX7 [https://www.rcsb.org/structure/2fx7]^13^). Coloring and inset as in **a**. **c** PGZL1–MPER_671-683_ combining site (wheat, LC; green, HC; pink, MPER; interacting residues - sticks). Shaded regions highlight aromatic clusters. **d** 4E10-MPER_671-683_ combining site, colored as in **c**. **e** Superposition of unbound (blue) and MPER_671-683_-bound (gray) H4K3. Ions are shown as sticks. **f** Superposition of unbound (yellow, CDR loops, brown) and MPER_671-683_-bound (gray) PGZL1 gVmDmJ. **g** CDR loops in bound (gray) and unbound (brown) PGZL1 gVmDmJ with residues that influence loop conformations shown as sticks. **h** Same region as in **g** for the mature PGZL1 structure.

To gain insight into PGZL1 three-dimensional (3D) structure and mode of binding, we determined crystal structures of unbound (1.4 Å resolution) and MPER_671-683_-bound PGZL1 (3.65 Å) Fabs (Fig. 4a, b and Supplementary Table 7). The superposition of Cα atoms of the two PGZL1 variable domains yielded a root means square deviation (r.m.s.d.) of 0.6 Å (Fig. 4a). Except for CDRH3 where residues are displaced by up to ~ 4.8 Å (i.e., W_99_), bound and unbound Fabs show only minor differences in CDR loops. PGZL1 resembles the 4E10 structure (PDB 2FX7 [https://www.rcsb.org/structure/2fx7]^13^) (Cα r.m.s.d. of ~ 0.4 Å; Fig. 4b). PGZL1 CDRH3 has a well-defined density, even in the absence of peptide, compared with 4E10’s three-residue longer CDRH3, where the residues G_99_WGW_100b_ at its tip were less defined. W_99_ in PGZL1 is ~ 7.8 Å apart from the equivalent W_100b_ in 4E10 (Supplementary Fig. 1a), neither of which contact MPER_671-683_ (Fig. 4b–d; PDB 2FX7 [https://www.rcsb.org/structure/2fx7]). 4E10 W_100b_ may contact the TM region of gp41^28^, but the shorter CDRH3 makes TM contact unlikely with PGZL1. However, F_100_ of PGZL1 CDRH3 (Fig. 4c), which corresponds to L_100c_ in 4E10, may re-orient to make aromatic interactions with MPER W_680_ and Y_681_ in the viral membrane.

PGZL1 binds to MPER_671-683_ using similar contact residues as 4E10 in CDRs H1, H2, H3, and L3 (Fig. 4c, d). In both complex structures, MPER_671-683_ is helical with W_672_-D_674_ in a capping 3_10_ helix, and germline-encoded Y_91_ (LC) and F_102_, W_47_ (HC) form an aromatic patch with peptide F_673_ and W_672_. Another aromatic patch is seen near MPER W_680_ and Y_681_, but the paratope residues differ between PGZL1 and 4E10. In the PGZL1 structure, F_100_ (L_100_ in 4E10) is close to these MPER aromatics. In the 4E10 structure, germline-encoded Y_32_ of CDRH1 (L_32_ in PGZL1) is also involved in this patch (Fig. 4c, d). The greater potency observed with 4E10 L_100c_F may be due to improved interaction of its CDRH3 with MPER and membrane by adding F_100c_ to this hydrophobic patch. Some hydrogen bonds between 4E10 and MPER residues are also observed in PGZL1, but the lower resolution of the PGZL1-MPER_671-683_ structure (3.65 Å) precludes a precise count.

### Unbound H4K3 is precisely preconfigured to bind MPER

Crystal structures of H4K3 unliganded and with MPER_671-683_ bound were determined at 1.45 Å and 1.98 Å resolution, respectively. Strikingly, their variable regions, including CDR loops, are in nearly identical conformations (Cα r.m.s.d. ~ 0.2 Å), which are both similar to the MPER-bound PGZL1 (Cα r.m.s.d. ~ 0.4 Å), suggesting H4K3 is already preconfigured for MPER binding (Fig. 4e). The shorter and more rigid CDRH3 of H4K3 may explain its lower polyreactivity compared with 4E10 whose CDRH3 is more flexible and hydrophobic.

PGZL1 and H4K3 structures closely resemble VRC42.01 structure (PDB 6MTO [http://www.rcsb.org/structure/6MTO]; Cα r.m.s.d. of the variable domains ~ 0.6 Å). However, a slight difference in MPER binding is observed at the N-terminal region of the epitope (residues 671–675; Supplementary Fig. 5a). All MPER epitope residues are in nearly identical positions when bound to PGZL1, H4K3, and 4E10 (Supplementary Fig. 5c, d). In contrast, the N-terminal region is shifted slightly away from the combining site in VRC42.01 (1.1 Å difference between the Cα of MPER F_673_ in PGZL1 and VRC42.01, and as much as 1.8 Å in the aromatic ring position; Supplementary Fig. 5b). It is noteworthy that the MPER peptide used in our crystal structures differs at position 677 from that in the VRC42.01 complex: asparagine vs. lysine, respectively (Supplementary Fig. 5b). This residue is located at the periphery of the combining site where the MPER position is similar in both structures. Thus, the different positioning of the N-terminal region of the MPER might be due to difference in residues of the J gene region between PGZL1 and VRC42.01 (Supplementary Fig. 1a and Supplementary Table 2). Phenylalanine at the position 100 g in the CDRH3 loops of PGZL1, H4K3, and 4E10 form an aromatic cluster with CDRL3 Y_91_ and MPER F_673_. In VRC42.01, this aromatic cluster is less tightly packed due to a methionine at CDRH3 position 100 g (Supplementary Fig. 5b-d).

### PGZL1 neutralization resistance mutations and MPER Ala scan

To identify resistance mutations to PGZL1, we first performed long-read NGS analyses on *env* rescued from contemporaneous PG13 donor plasma. Sequences were determined for 58 clade B Envs. One small sublineage and a larger, more diverse clade were predicted to be CXCR4 tropic and CCR5 tropic, respectively (Supplementary Fig. 6a). Notably, MPER polymorphisms D_674_S, D_674_T, and D_674_E are predicted to contact PGZL1 and are distinct from other reported 4E10 resistance mutations, F_673_L or W_680_G/R^29,30^ (Supplementary Fig. 6a). As viruses pseudotyped with PG13 Envs lacked infectivity, we tested the MPER polymorphisms in the context of primary isolate COT6, while also testing PGZL1 and 4E10 against a COT6 MPER Ala mutant virus panel. Indeed, D_674_S, D_674_E, and D_674_T, all made COT6 more resistant to neutralization by PGZL1, H4K3, and 10E8 (Table 1). Mutant D_674_A was resistant to PGZL1 and H4K3, but less so with 4E10. Some PG13 Envs have Gly at MPER position 662, which is present in 1.2% (vs. Glu in 71.7%) of Envs in the LANL database (Supplementary Fig. 6c). However, A_662_G resulted in COT6 being tenfold more sensitive to PGZL1 and 4E10 (Supplementary Fig. 6b). All COT6 mutants were similarly sensitive to control antibody VRC01. Overall, we infer that some viruses in the PG13 donor developed polymorphisms in D_674_ to resist PGZL1 antibodies.

**Table 1:** Effect of MPER mutation on the neutralization of HIV COT6 by PGZL1 and control antibodies.

Virus	MPER epitope	COT6 mutants	Fold increase in IC_50_				
			PGZL1	H4K3	4E10	10E8	VRC01
COT6	SWFDITKWLW	WT	1	1	1	1	1
PG13 Cons	NWFDITNWLW		NA	NA	NA	NA	NA
PG13 isolate 1	NWFSITNWLW	D674S	7.7	6.29	1.65	NA	NA
PG13 isolate 2	NWFEITNWLW	D674E	26	12	10	17.5	1.2
PG13 isolate 3	NWFTITNWLW	D674T	120	38	25	36.0	1.0

*NA*, not attempted

COT6 Ala mutants W_672_A, F_673_A, and W_680_A were resistant to PGZL1, HK43, and 4E10 (Supplementary Fig. 6c), as well as to 10E8^31,32^. However, MPER Ala mutations rarely occur naturally (Supplementary Fig. 6c). Fold changes in IC_50_ differed among the 4E10-like bnAbs against mutants S_671_A, D_674_A, I_675_A, and L_679_A (Supplementary Fig. 6b). Thus, IC_50_s of H4K3 and 4E10 against S_671_A and L_679_A differed by up to 100-fold, despite a cross-clade correlation in their IC_50_s (Supplementary Fig. 3d). In H4K3 and 4E10-bound forms, S_671_A has a similar structural environment, so this mutation might impact MPER folding or accessibility. However, neutralization differences with COT6 L_679_A seem more likely to be due to HC position 54, which is occupied by a valine, phenylalanine, and leucine in H4K3, PGZL1, and 4E10, respectively; structural analysis of this region reveals more tightly packed interactions between the MPER and 4E10 vs. PGZL1 or H4K3. Of note, MPER mutations L663A, D664A, S665A, W666A, K667A, L669A, W670A, K677A, and W678A enhanced HIV neutralization by the three MPER bnAbs, as also reported previously for 4E10^32^. The neutralization enhancement effect with these mutants is not fully understood but might be explained by changes in Env conformation that increase accessibility and/or susceptibility to functional inactivation by the MPER bnAbs^31^.

### PGZL1 inferred germline structure

The MPER_671-683_-bound (2.47 Å) and -unbound (2.6 Å) structures of inferred germline PGZL1 gVmDmJ notably differ in the conformation of their CDRs L1, L3, and H3 (Fig. 4f). When superimposed, residues in CDRL3 in the unbound structure are shifted up to 6.8 Å from those in the bound complex. This shift of CDRL3 in the bound structure may arise from MPER binding, which cause a rearrangement in the aromatic interaction between Y_32_ (CDRL1) and Y_91_ (CDRL3) (Fig. 4g). We note that Y_32_ has affinity matured in PGZL1 to alanine, which does not affect Y_91_’s orientation; thus, the CDRL3 and L1 conformations are similar in both unbound and bound PGZL1, and resemble the bound form of the inferred germline PGZL1 Fab (Fig. 4h).

### Structural and functional evidence of PGZL1 membrane binding

**Fig. 5:**
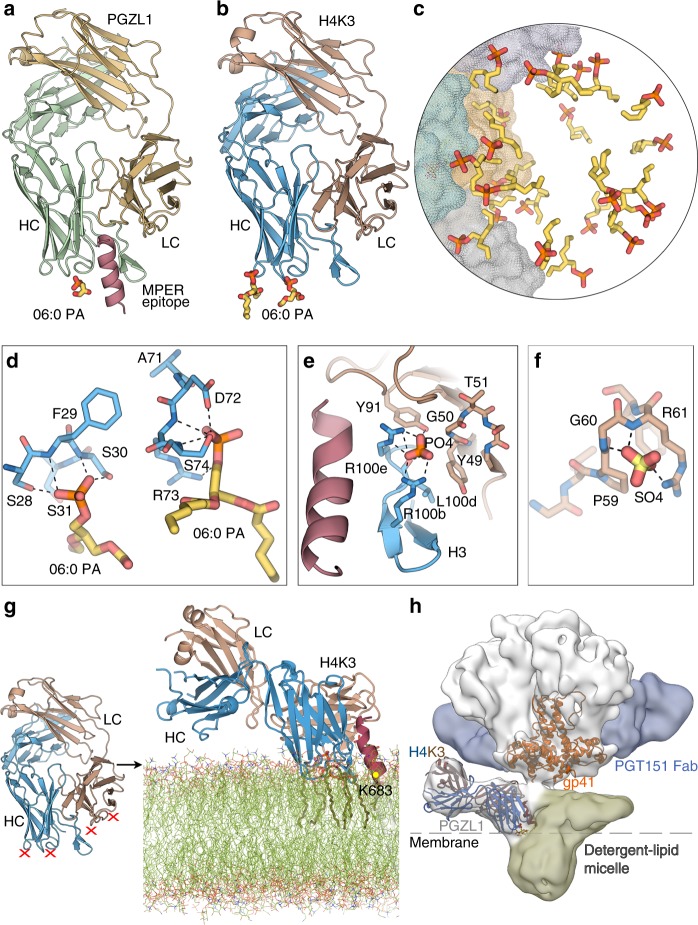
Lipid binding and angle of approach of PGZL1 variants to the viral membrane. **a** Cartoon of the PGZL1-MPER_671-683_ complex structure (wheat, LC; green, HC; pink, MPER) crystallized with 06:0 PA (sticks). **b** Cartoon of H4K3 (brown, LC; blue, HC) crystallized with 06:0 PA (sticks). **c** Stick rendering of 06:0 PA fragments forming a lipid vesicle at the interface of 12 crystallographic and non-crystallographic-related H4K3 Fabs. The four Fabs in the asymmetric unit are shown as gray, green, yellow, and blue color surfaces. **d** Stick rendering of observed lipid-binding sites in H4K3. **e** Phosphate-binding site near to H4K3 CDRH3 when MPER_671-683_ (pink) is bound. Colors as in **b**. **f** Sulfate-binding site in FRL3 of H4K3. **g** Model of H4K3 binding to the MPER (red)-viral membrane (green) epitope. The model at the right side of the arrow was built based on the regions where experimental lipids and anions (red X) bind on H4K3 (left side of the arrow); cognate lipids are shown as sticks inside the modeled membrane. The position of the MPER K683 residue is indicated with a yellow dot. **h** Cryo-EM reconstruction of full-length AMC011-PGT151-PGZL1 complex at 8.9 Å with H4K3 (blue/brown ribbons) fitted into the Env density at the base of the gp41 stem. The MPER is shown as a red ribbon and lipid head groups as sticks. The detergent–lipid micelle is shown in olive and PGT151 density in blue. Dashed lines show the approximate location where the outer surface of the membrane would be on the virus or infected cells.

To elucidate potential membrane interactions of PGZL1, we solved crystal structures of PGZL1-MPER_671-683_ and unliganded H4K3 in the presence of a short acyl tail phosphatidic acid (06:0 PA) at 3.42 Å and 3.11 Å resolution, respectively. We observed lipid-binding sites in both antibody structures (Fig. 5a, b). The first lipid site in PGZL1 and H4K3 is in a similar location in 4E10^6^, proximal to CDRH1 S_28_, F_29_, and S_30_ (Fig. 5d and Supplementary Fig. 7a). Next to this site in H4K3, a second PA lipid interacts with D_72_, R_73_, and S_74_ in the HC framework region FRH3. Of note, in H4K3, these 2 lipids are part of a ~ 33 Å lipid vesicle formed at the interface of 12-symmetry-related Fabs that each contain the 2 lipid sites (Fig. 5c), as also observed in the 4E10 structure^6^.

**Fig. 6:**
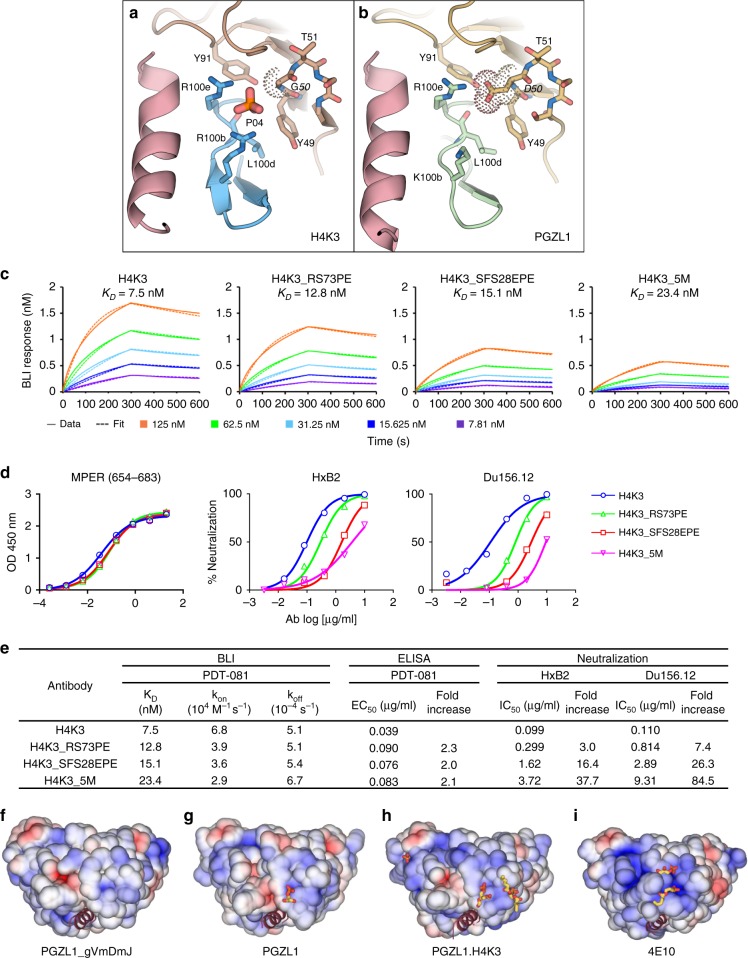
MPER-induced PO_4_-binding site of H4K3 and PGZL1 lipid site mutant characterization and electrostatics. **a**, **b** Stick rendering of the PO_4_-binding site in (**a**) H4K3 and (**b**) PGZL1. The side chain at LC position 50 in each antibody is surrounded by dots. Select HC residues of the two antibodies are shown in blue (**a**) and green (**b**), and the LC is shown in brown. MPER is shown as a pink ribbon. **c** Binding of H4K3 lipid-binding site mutants to immobilized MPER peptide by BLI. **d** Binding of H4K3 lipid-binding site mutants to MPER peptide by ELISA and neutralization (log IC_50_) of HxB2 and Du156.12. **e** BLI, ELISA binding to MPER peptide, and neutralization statistics. **f**–**i** Surface rendering, along the MPER helical axis (red ribbon), of the solvent accessible electrostatic potential contoured at ± 5 kT/e for (**f**) PGZL1 gVmDmJ, (**g**) PGZL1, (**h**) H4K3, and (**i**) 4E10. Observed lipid fragments and anions are shown as sticks. Source data for **h** and **i** are provided as a Source Data file.

PO_4_ and SO_4_ ions were both bound at the CDRH1 site of the H4K3 structures obtained in lipid-free buffer (Fig. 4e). Another PO_4_ ion interacts with R_100b_ and R_100e_ of CDRH3 and Y_91_ of CDRL3 in the H4K3-MPER_671-683_ structure (Figs. 4e and 5e, and Supplementary Fig. 7b). As this anion-binding site is absent in peptide-free H4K3, despite the crystallization buffer containing SO_4_, this site may be induced by MPER binding. This site is also H4K3 specific, as it is absent in peptide-bound PGZL1, where LC SHM-residue D_50_ replaces G_50_ (Fig. 6a, b) and residue 100_b_ is lysine. Another SO_4_ ion was observed in unbound H4K3 proximal to FRL3 and interacts with P_59_, G_60_, and R_61_ (Fig. 5f and Supplementary Fig. 7c). Thus, these two anion-binding sites may also represent phospholipid-binding sites.

To determine whether the lipid-binding sites observed in our structures are biologically relevant, we mutated the H4K3 lipid-binding sites. Both CDRH1 and FRH3 regions form plateau-like structures with main-chain nitrogens making contacts with the lipid head groups (Fig. 5a, b, d, f). We thus mutated the lipid-binding regions S_28_F_29_S_30_ of CDRH1 and D_72_R_73_S_74_ of FRH3 to E_28_P_29_E_30_ and D_72_P_73_E_74_, respectively, with the proline mutation chosen to perturb the lipid interaction with the main chain and the glutamate mutation to introduce negative charges that are repulsive to the viral membrane. We also mutated residue G_50_ of the H4K3 LC to D_50_, to disrupt the CDRH3 R_100b_-R_100e_ site. In summary, we created the following three mutants: (1) H4K3_SFS28EPE that includes E_28_P_29_E_30_ in the HC and D_50_ in the LC; (2) H4K3_RS73PE that includes P_73_E_74_ in the HC and D_50_ in the LC; and (3) H4K3_5M that includes all five mutations in the HC combined with D_50_ in the LC. ELISA and BLI experiments show that the three mutants retain similar nM binding affinity to the MPER peptide as H4K3 (Fig. 6c, e). However, the neutralization potency (IC_50_) against HxB2 and Du156.12 is reduced with all three mutants by 7–84-fold, with the H4K3_SFS28EPE and H4K3_5M exhibiting higher losses in potency (Fig. 6d, e). Hence, mutation of the lipid-binding sites influences binding to membrane-embedded Env on the virus but not binding to MPER out of the membrane context.

Analysis of the surface potential of PGZL1 variants and 4E10 show that the lipid head groups and anions bind to electropositive clefts on the antibody surface (Fig. 6f–i). Germline PGZL1 gVmDmJ and mature PGZL1 have less basic patches compared with H4K3 and 4E10. Thus, their lower neutralization potency may correlate with reduced electrostatic interaction with specific lipids in the viral membrane.

Considering the position and orientation of lipids, anion-binding sites (Fig. 5b–g), and MPER orientation (Fig. 4e), we generated a molecular model of H4K3 binding to the MPER-viral membrane using CHARMM force field and molecular dynamics (MD) simulation, in a similar way to the published 4E10 and 10E8 models^6,33,34^. The lipid head groups of the membrane outer leaflet were placed in a plane that roughly includes K_683_ of gp41 as well as the two lipid head groups and anion sites observed in our crystal structures (Fig. 5g). Our model suggests that H4K3 contacts the membrane-MPER epitope in a similar manner to 4E10^6^ with the MPER helix tilted 67°–73° from the bilayer surface. CDRH1, CDRH3 tip, FRH3 (residues 73–76), N-terminal HC residue K_1_, and CDRL2 and FRL3 (residues 56–61 and R_77_) are proximal to and interact with the lipid heads. A hydrophobicity plot showed similar membrane-transfer propensity between PGZL1, H4K3, VRC42.01, and 4E10 in both HCs and LCs, except that VRC42.01 and 4E10 CDRH3 showed higher hydrophobicity (Supplementary Fig. 1b).

### Cryo-EM structure of Env-PGT151-PGZL1 complex

To explore the topology of the PGZL1-Env interaction, we obtained a cryo-EM reconstruction at 8.9 Å resolution of a full-length Env of isolate AMC011^35^ in complex with PGZL1 and PGT151. Despite notable structural heterogeneity between the well-resolved ectodomain vs. detergent–lipid micelle containing the TM and cytoplasmic tail, we classified a subset of particles and reconstructed a 3D map with one PGZL1 Fab bound to one MPER of the AMC011 trimer (Fig. 5h and Supplementary Fig. 6). Two PGT151 Fabs were also observed, whereas the gp41 TM region cannot be distinguished from the detergent–lipid micelle. Fitting of the PGZL1 lipid-bound Fab crystal structure (Fig. 5g) into the cryo-EM map confirms the antibody orientation relative to the bilayer and suggests both HC and LC contact the membrane (Fig. 5h). The angle of approach revealed in the MD simulation (Fig. 5g) also concurs with the cryo-EM docking model.

## Discussion

HIV bnAbs that share epitope and paratope features have been of increasing interest for vaccine design^2^. The bnAbs 4E10, 10E8, and DH511 show near pan-neutralization of HIV-1 by targeting nearly identical α-helical epitopes in the MPER, but these antibodies have uncommon features. We described PGZL1 and its lineage variant H4K3 and germline revertants that have some common features that may aid vaccine design, especially given the homology to 4E10 and the recently described VRC42 antibody. PGZL1 antibodies have a 15-residue CDRH3 that is close to the average in humans of ~ 13.2^7^. Similar to all MPER bnAbs, CDRH3 aromatics were crucial for HIV neutralization, but the observed pre-configuration of H4K3 to bind the MPER and lipid heads on the membrane may explain its faster on-rate compared with PGZL1, where reorientation of the CDRH3 is needed. An induced lipid site at residues R_100e_/R_100b_ of H4K3 may also have improved its potency while reducing polyreactivity. By comparison, longer CDRH3s on 4E10 and VRC42 variant VRC42.N1 may interact more with the TM domain in the upper membrane core^28^. Using X-ray crystallography and cryo-EM, we determined the orientation, angle of approach, and composition of the PGZL1-Env interaction. These valuable data can inform the design of HIV vaccines and therapies, involving a vulnerable site that has long precluded a complete description due to ambiguity of the membrane interaction.

PGZL1, 4E10, and VRC42 are derived from similar V/D genes but different J genes. Whether other shared CDRH3 residues arose from SHM or N1/N2 additions is unknown but should be considered, as they may affect antibody activity. Vaccine design must also consider how to elicit bnAbs with few SHM. Fortunately, the SHM-reverted PGZL1 gVmDmJ antibody neutralized up to 28% of viruses tested, albeit mostly with limited potency; many somatic mutations in 4E10 and 10E8 are also non-essential^18,19^. *V*_*H*_*1-69* is also highly mutable due to several SHM hotspots^36^. *V*_*H*_*1-69* has been associated with autoantibodies of leukemia^36^, HCV-associated mixed cryoglobulinemia^37^, as well as bnAbs highly specific to the influenza hemagglutinin stem^38^ and HCV E2^39^. Thus, any vaccine strategy to elicit *V*_*H*_*1-69* MPER bnAbs must be MPER specific and avoid or limit activation of off-target B cells.

We found a few Envs that were recognized and neutralized by germline revertants of PGZL1 and 4E10. Such Envs, or perhaps the T/F Env of the VRC42 donor^5^, might be useful for activating 4E10-like B-cell precursors whose CDRH3s can engage membrane-bound Env. These B cells could be boosted with a heterologous Env to drive the maturation process required for achieving neutralization breadth and potency (Pathway 1). Alternatively, *V*_*H*_*1-69* restricted B cells that lack crucial lipid-interacting residues might be activated, perhaps using specific Envs, scaffolds, peptides, or anti-idiotype antibodies^3,40–42^. Lipid-interacting residues would have to be provided by the maturation process and the B cells boosted perhaps by a membrane-anchored MPER (Pathway 2). Contrarily, 10E8 and DH511 do not bind Env as germline revertants; thus, specific strategies may be needed to activate such lineages. As soluble Env differs somewhat from its membrane counterpart in glycosylation and conformation^43–45^, development of membrane-embedded Env vaccines or suitable soluble alternatives seems justified^25^.

H4K3 lacks the potency of bnAbs currently being developed for therapy, such as 10E8^10^. However, its exceptional breadth warrants efforts to engineer H4K3 to be more potent without enhancing polyreactivity, as was successfully accomplished recently with 10E8^46,47^.

MPER bnAbs have been associated with IgG3-prone B-cell subsets^4^. PGZL1 shows that IgG1 MPER B cells can also be elicited in humans, which we speculate arose from a precursor with minimal polyreactivity that bound directly to Env. Non-IgG3 MPER nAbs have notably also been observed in donor plasma^48^ and variants of VRC42 are both IgG1s and IgG3s. Whether such differences in isotype, polyreactivity, or autoreactivity of 4E10-like bnAbs translate into useful correlates for eliciting MPER bnAbs requires future immunization studies.

## Methods

### Ethics statement, study participant, and samples

Donor PG13 was from the IAVI-sponsored Protocol G cohort in South Africa^20^. Blood samples were collected with written, informed consent, and the study was reviewed and approved by the relevant Ethics and Research Committees.

### Isolation of PGZL1 monoclonal antibodies

Fluorescent-labeled antibodies CD3-Alexa Fluor 700 (BD 557943), CD8-Alexa Fluor 700 (BD 561453), CD14-PE-Cy7 (BD 561385), CD16-PE-Cy7 (BD 560716), CD19 PerCP*Cy5.5 (BD 561295), CD20 PerCP*Cy5.5 (BD 350955), IgG-PE-Cy5 (BD 551497), IgM-BV605 (BD 562977), and IgD-BV605 (BD 563313), which target cell surface markers, were used as 1:200-fold dilution in staining. Biotin-labeled MPER peptide PDT-081 (E_654_KNEQELLELDKWASLWNWFDITNWLWYIK_683_-biotin) was purchased from GenScript and coupled separately to streptavidin-BV421 (BD 562426) and streptavidin-APC (allophycocyanin, BD 555335). PBMCs were stained using the LIVE/DEAD Fixable Near-IR Dead Cell Kit (Life Technologies, L34957) for 30 min on ice. Cells were then labeled with antibodies cocktail along with MPER probes for 1 h in Brilliant Staining buffer (BD 563794) on ice. Cell population CD19+/CD20+, CD3−/CD8−, CD14−/CD16−, IgG+, IgD-/IgM− MPER double positive were sorted using BD FACSAria III sorter into individual wells of a 96-well plate containing lysis buffer and plates were immediately sealed and stored at −80 °C. The single B-cell sorting strategy is shown in the Supplementary Fig. 9.

### Antibody sequence amplification, analysis, and cloning

The first-strand complementary DNA from B cells was synthesized using Superscript III Reverse Transcriptase (Life Technologies) and random hexamers (Gene Link). Nested PCR amplification of HC and LC variable regions was performed using Multiplex PCR Kit (Qiagen). Amplified HC and LC variable regions were sequenced and then analyzed using IMGT online tools. Antibodies of interest were cloned into expression vectors by re-amplification of the variable regions using the same primers but modified to introduce homology to the vector^49^. Primers used are reported in the Supplementary Table 8.

### PG13 antibody repertoire sequencing and analysis

The 5′-RACE (rapid amplification of cDNA ends) PCR protocol used for unbiased human B-cell repertoire analysis has been previously described^22,50^. Briefly, total RNA was extracted from fivemillion PBMCs into 30 μl of water with RNeasy Mini Kit (Qiagen). 5′-RACE was performed with SMARTer RACE cDNA Amplification Kit (Clontech). The immunoglobulin PCRs were set up with Platinum Taq High-Fidelity DNA Polymerase (Life Technologies) in a total volume of 50 µl, with 5 μl of cDNA as template, 1 μl of 5′-RACE primer, and 1 μl of 10 µM reverse primer. The 5′-RACE primer contained a PGM/S5 P1 adaptor, whereas the reverse primer contained a PGM/S5 A adaptor. A total of 25 PCR cycles were performed and the expected PCR products ( ~ 600 bp) were gel purified (Qiagen). NGS was performed on the Ion S5 system as previously described^21^. Briefly, heavy (H), kappa (κ), and lambda (λ) chain libraries were quantified using Qubit® 2.0 Fluorometer with Qubit® dsDNA HS Assay Kit, and were mixed at a ratio of 2:1:1 before antibody libraries from three time points were further mixed at a ratio of 1:1:1. Ion Xpress^TM^ barcodes (Life Technologies), #1–#3, were used to tag antibody libraries to differentiate the three time points. Template preparation and (Ion 520) chip loading were performed on Ion Chef using the Ion 520/530 Ext Kit, followed by sequencing on the Ion GeneStudio S5 platform with default settings. Raw data were processed without the 3′-end trimming in base calling to extend the read length. An improved version of the Antibodyomics pipeline^21^ was used to process, annotate, and analyze the sequencing data of PG13 antibody repertoires. After pipeline processing, a bioinformatics filter was applied to remove erroneous sequences that may contain swapped gene segments due to PCR errors. Specifically, a full-length variable region sequence would be removed if the V-gene alignment was <250 bp. The results for pipeline processing are summarized in Supplementary Table 3. The pipeline-processed antibody chain sequences were subjected to two-dimensional (2D) divergence/identity analysis and CDR3-based lineage analysis, with putative somatic variants determined at CDR3 identity cutoffs of 80% and 95% (Fig. 1d).

### Expression and purification of PGZL1 IgG and Fab variants

PGZL1 HCs and LCs were cloned using Gibson Assembly Enzyme mix (NEB) into expression vectors with the appropriate IgG1, Igκ, or Igλ constant domains^49^. Antibodies were expressed in FreeStyle 293F cells (Life Technologies Cat#R79007). Briefly, ~ 750 µg DNA (500 µg HC and 250 µg LC plasmid) were added to 25 ml Opti-MEM (Life Technologies, 31985-070), which was mixed with Opti-MEM containing 2250 µg polyethylene imine MAX (molecular weight 40,000 kDa; Polyscience, 24765-1). After incubation for 20 min at room temperature (RT), the transfection mix was added to 1 L cells at a density of ~ 1.2 × 10^6^ cells/ml in FreeStyle293 Expression Medium (Life Technologies, 12338018). The cells were incubated at 37 °C and 8% CO_2_ for 6 days. After collecting the cells, the supernatant, containing IgG or Fab, was filtered and loaded into a protein A beads column (Thermo Scientific) or HiTrap KappaSelect column (GE Healthcare Life Sciences, 17545812). The column was washed with phosphate-buffered saline (PBS) and eluted with 0.2 M citric acid pH 3.0 or 0.1 M glycine pH 2.7. The fractions were concentrated and the buffer was changed to 20 mM sodium acetate pH 5.5. The Fab was loaded into a Mono S column and was eluted with a 0–60% linear gradient of 1 M sodium chloride and20 mM sodium acetate pH 5.5 buffer. The Fabs were concentrated and stored in 20 mM sodium acetate pH 5.5 at 4 °C.

### Crystallization of the PGZL1 Fab variants

All crystal trials were performed with our Scripps/IAVI/JCSG high-throughput CrystalMation robot (Rigaku) using protein sample (unbound or peptide-bound Fab) at ~ 7 mg/ml. Crystals were obtained using sitting drop vapor diffusion by mixing a 1:1 protein:reservoir solution in 200 nl drops. The MPER_671-683_ peptide sequence used for the Fab complexes was N_671_WFDITNWLWYIK_683_-KKK. Fab-MPER_671-683_ complexes were prepared by mixing Fab with peptide in a 1:5 protein:peptide molar ratio. PGZL1-MPER_671-683_ and H4K3 were co-crystallized with 06:0 PA by mixing highly concentrated protein sample with 06:0 PA (stock solution of 15 mM in 20 mM sodium acetate, pH 5.5) such that the final concentrations of the protein and lipid in the mixture were ~ 7 mg/ml and ~ 8 mM, respectively. The crystallization conditions and cryo-protectant are reported in Supplementary Table 7.

### Data collection, structure determination, and refinement

X-ray diffraction data sets were collected at SSRL on the 9-2 or 12-2 beamlines (Supplementary Table 7). The data sets were processed using HKL2000^51^ or XDS^52^. Phaser^53^ was used to find molecular replacement solutions employing the 4E10 Fab variable and constant domains (PDB 2FX7 [https://www.rcsb.org/structure/2fx7]) as the search model. After an initial round of rigid body refinement, model rebuilding was carried out with Coot^54^ and refinement with Phenix^55^ using different refinement strategies as appropriate for the resolution of each structure. Final statistics are summarized in Supplementary Table 7. Structural images were generated using PyMOL (The PyMOL molecular graphics system).

### MD model of H4K3 interaction with MPER epitope on membrane

A trimeric model of MPER epitope-gp41 TM region was constructed as previously described^6^ using PDB 2MOM [https://www.rcsb.org/structure/2mom] as a template. Briefly, residues 379–403 of LAMP-2A TM region were replaced with the gp41 TM region [residues 686–710; (UNIPROT ID: Q70626, HIV-1-LW123 numbering)], such that Arg796 on gp41 is located in the TM inter-helical interface. The orientation of the MPER in this model is based on the MPER_671-683_ and lipid orientation observed in our crystal structures. The H4K3 Fabs and the 06:0 PA lipids fragments were added to the model by superposing the MPER_671-683_ to the same region of the model. The acyl tails of the crystallographic lipids were extended to the size of a 1,2-dipalmitoyl-sn-glycero-3-phosphate (DPPA) molecule and the SO_4_ and PO_4_ ions bound to CDRH3 and FRL3 regions were replaced with DPPAs with the lipid tails pointing in the same direction as those of the lipids observed in the structures. Thus, the lipid tails are oriented approximately perpendicular to the plane that roughly includes the observed lipid head groups, two anions and Lys683 of the MPER. The lipid bilayer was placed with CHARMM^34^ on the putative TM region built to anchor in the membrane. The replacement method was used to place 424 and 455 lipids in the upper and lower leaflet, respectively, of a heterogenous lipid bilayer in a rectangular box of *x* = *y* = 161.2 Å. The membrane composition of the heterogenous bilayer was chosen based on the HIV-1 membrane lipid composition^56^. Potassium counter ions were placed with the Monte-Carlo method. In the final model, the head group of the lipids corresponding to the sites observed in the crystal structure and the Lys_683_ of MPER are located within the head group region of the membrane outer leaflet.

### Full-length Env sample preparation for cryo-EM

Recombinant full-length AMC011 Env was expressed in HEK293F cells and purified as described previously^45,57^. Briefly, HEK293F cells (density of 1.6 × 10^6^ cells/ml; Thermo Fisher Cat#R79007; RRID: CVCL_D603) were transfected with furin and Env-encoding plasmids at 1:3 furin:Env ratio using PEImax. Cells were collected ~ 3 days post transfection, washed once with 400 ml/l cold PBS, and incubated with PGT151 TEV IgG (containing a TEV protease cleavage site between the Fc and the Fab regions) in lysis buffer containing 0.5% v/v Triton X-100, 50 mM Tris pH 7.4, 150 mM NaCl. After removal of cell debris by centrifugation, the supernatant was incubated with Protein A resin (GBiosciences) overnight. Resin was then transferred to gravity flow column, washed, and exchanged to buffer containing 50 mM Tris-HCl pH 7.4, 150 mM NaCl, 0.1% (w/v) DDM (N-Dodecyl-Beta-D-Maltoside), 0.03 mg/ml deoxycholate, and 2 mM EDTA prior to elution by addition of 0.25 mg of TEV protease per liter of initial HEK293F. The complex was further purified using a Superose 6 column (GE Healthcare) using the same buffer excluding EDTA. Purified protein was concentrated to 5.6 mg/ml prior to cryo-EM grid preparation. Three microliters of PGZL1 Fab at 4.5 mg/ml and 1 µl of 1 mM lipid mix (DOPC:DOPS:CHS:PIP2 at 40:40:16:4 molar ratio, Avanti Polar Lipids) was added to 10 µl of purified and concentrated AMC011 Env. Detergent removal was initiated by three additions of ~ 3–5 SM-2 bio beads (Bio-Rad) with 1 h incubation between each addition. After the last incubation, 3 µl of sample was applied to either plasma cleaned 1.2/1.3 C-Flat Holey Carbon grid (Protochips) with 0.5 µl of 0.01% amphiphol A8-35 or to 2/2 Quantifoil Holey Carbon Grid with 0.5 µl of 35 µM LMNG. Amphiphol or LMNG was added directly on grid to improve orientation distribution of particles. Grids were plunge-frozen in liquid ethane using Vitrobot mark IV (Thermo Fisher Scientific) without wait time, blot force of 0 and 7 s blot time.

### Cryo-EM data collection and processing

Micrographs (5775) were collected using our Titan Krios (Thermo Fisher Scientific) operating at 300 keV and K2 Summit direct electron detector (Gatan). Data were collected with Leginon automated image acquisition software^58^ at ×29,000 magnification resulting in pixel size of 1.03 Å in the specimen plane. Forty-six frames were collected for each micrograph with 250 ms exposure time per frame at dose rate of 4.7 e^−^/pix/s and with defocus values ranging from −1.0 to −2.5. Frames were aligned and dose weighted with MotionCor2^59^ and CTF models for each micrograph were calculated using GCTF^60^. Workflow for subsequent data processing is presented in Supplementary Fig. 8. Particles were picked using DoGPicker^61^ and, after initial round of 2D classification in CryoSPARC^62^, 356,587 Env particles were moved to Relion^63^ for 3D processing. After two rounds of 3D classification, a class of 15,214 Env particles with one PGZL1 Fab bound and two PGT151 Fabs bound showed the best Fab definition and was refined to 8.9 Å resolution according to the FSC 0.143 gold-standard criterion.

### HIV-1 neutralization assays

Neutralization activity of PG13 donor plasma and monoclonal antibodies was assessed using pseudovirus and a single round of replication in TZM-bl target cells (NIH AIDS Reagent Program; Cat#8129-442, RRID: CVCL_B478). Pseudoviruses were generated by co-transfection of HEK 293T cells (ATCC, Cat#CRL-3216, RRID: CVCL_0063) with an Env-expressing plasmid and an Env-deficient genomic backbone plasmid (pSG3ΔEnv). Pseudoviruses were collected 48–72 h post transfection and stored at −80 °C. Serial dilutions of plasma/antibody were incubated with virus in presence of DEAE-dextran and the neutralizing activity was assessed by measuring luciferase activity after 48–72 h. Dose–response curves were fitted using nonlinear regression to determine IC_50_ values. For competition assays, plasma/antibody dilutions were pre-incubated 30 min at RT in the presence or absence of 10 μg/ml of MPER peptide.

### Enzyme-linked immunosorbent assay

Half-area, 96-well ELISA plates were coated overnight at 4 °C with 50 μL PBS containing 250 ng of antigens per well. The wells were washed four times with PBS containing 0.05% Tween 20 and blocked with 4% non-fat milk (NFM) for 1 h at 37 °C. Serial dilutions of sera/antibodies were then added to the wells and the plates were incubated for 1 h at 37 °C. After washing four times, the wells were treated with goat anti-human IgG Fc conjugated to horseradish peroxidase (HRP) (Jackson ImmunoResearch, Cat#109-035-098), diluted 1:1000 in PBS containing 0.4% NFM and 0.05% Tween 20. The plates were incubated for 1 h at 37 °C, washed four times, and developed by adding HRP substrate diluted in alkaline phosphatase staining buffer (pH 9.8), according to the manufacturer’s instructions. The optical density at 405 nm was read on a microplate reader (Biotek Synergy). EC_50_ values were calculated using Prism6 (GraphPad).

### HEp-2 assay

Antibodies were assayed for autoreactivity using a HEp-2 indirect immunofluorescence kit (Bio- Rad) according to the manufacturer’s instructions.

### Antibody binding by biolayer interferometry

An Octet RED96 system (FortéBio) with BLI was used to assess the binding of PGZL1 and its variants Fab to MPER peptide PDT-081. Biotinylated peptide at 7.5 μg/ml in PBS/0.002% Tween 20/0.01% bovine serum albumin was captured on the surface of Streptavidin biosensors (FortéBio) for 8 min. The biosensor was exposed to a serial dilution of Fabs for 5 min and then to buffer for 5 min, to acquire association and dissociation sensograms, respectively. *K*_D_ values were calculated as *k*_off_/*k*_on_ based on five sensograms from the dilution series with a minimum *R*^2^ value of 0.99. The sensograms were corrected using the blank reference and fitting was accomplished using the FortéBio Data Analysis 7 software package.

### Flow cytometry

Comb-mut V4 cell line was generated and characterized as described^25^. Following a similar protocol, the MPER-TM_654-709_ cell line was developed. Briefly, a PCR amplicon encoding a TPA leader sequence, codon-optimized MPER-TM (654-709) of BG505, followed by a stop codon was cloned into NotI and XhoI sites of pLenti-III-HA (Applied Biological Materials). The resulting MPER-TM lentiviral vector was used to generate lentiviral particles, which were then used to transduce 293T cells following the manufacturer’s instructions. The transduced cells were cultured in medium containing 10 μg/ml puromycin to select for stable integrants and were sorted on a BD Aria flow cytometer to select the 10E8 (high) stable cell line MPER-TM_654-709_.

For flow cytometry experiments, a total of 10^7^ cells of stable cell lines HIV-1 MPER-TM_654-709_ or Comb-mut Env (V4) were washed in PBS and labeled with Fixable Aqua Dead Cell Stain (Life Technologies). Cells were washed in FACS buffer (PBS supplemented with 2% heat-inactivated fetal bovinse serum) and were stained with monoclonal antibody. After another wash, cells were stained using APC-conjugated mouse anti-human Fc (BioLegend HP6017). Soluble CD4 was incubated with cells for 30 min prior to staining cells with antibody. Cells were acquired and analyzed by using NovoCyte (ACEA Biosciences). Data were analyzed using FlowJo software (Tree Star).

### BN-PAGE mobility shift assay

Virus samples were pre-incubated with Fab fragments of antibodies for 30 min at RT. Samples were then solubilized with 1% DDM for 20 min on ice. Env was separated using BN-PAGE and was detected by western blotting using a cocktail of gp120 and gp41 antibody probes. Western blottings were imaged using a Chemidoc XRS (Bio-Rad) and analyzed using Image Lab software (Bio-Rad). The center of intensity for each Env trimer band and the distance that it had migrated along the gel was calculated. The relative shift of each band was calculated by setting the maximum shifted band in each experiment to 1, which corresponds to threefold occupancy of Fab per trimer and the antibody-free control to 0. The trimer occupancy at each Fab concentration was determined by calculating the relative shift for control Fabs PGT126 (3 Fabs/trimer), PGT151 (2 Fabs/trimer), and PG9 (1 Fab/trimer).

### Site-directed mutagenesis

Mutagenesis was performed using a Quikchange site-directed mutagenesis kit (Agilent Technologies).

### Lipid insertion propensity

Lipid insertion propensity scores were calculated using the MPEx (Membrane Protein Explorer) software as the sum of ΔGwif, the free energy of transfer of an amino acid from water to POPC (1-palmitoyl-2-oleoyl-glycero-3-phosphocholine) interface, over all amino acids of the antibody variable heavy and light domains.

### Statistical analysis

For all mAb/serum pseudovirus neutralization and ELISA assays, the IC_50_ or concentration of mAb/dilution of serum needed to obtain 50% neutralization against a given pseudovirus was calculated from the linear regression of the linear part of the neutralization curve. For neutralization assays in which a fold change in IC_50_ imparted by a virus mutant or virus treatment was reported, the IC_50_ obtained for one virus/assay condition was divided by the IC_50_ obtained for the other virus/assay condition, as indicated in the figure legends. Two-way analysis of variance was used for multiple group comparison.

### Full-length Env amplification sequencing and analysis

HIV-1 Env was sequenced as described^49^. Briefly, virions were purified from plasma through a sucrose cushion and ultracentrifugation. RNA was extracted (Viral RNA Mini Kit, Qiagen) and reverse transcribed (SuperScript III, Thermo Fisher). PCR was performed with subtype B primers using 45 PCR cycles. Four replicate PCR reactions were pooled, purified (QIAquick, Qiagen), visualized, and quantified (2100 Bioanalyzer System, Agilent Biosciences). Preparation and sequencing of SMRTbell template libraries of ~ 2.6-kb insert size were performed according to the manufacturer’s instructions (Pacific Biosciences) using P6/C4 chemistry on the RS-II.

CCS sequences were constructed using the PacBio SMRTportal software (version 2.3). The Robust Amplicon Denoising algorithm^64^ was used for error correction and MAFFT^65^, with manual curation, was used to construct a multiple sequence alignment. Phylogenies were reconstructed using FastTree v2.1 and were visualized with FigTree. Geno2Pheno 2.5^66^ was used to predict co-receptor tropism.

### Reporting summary

Further information on research design is available in the Nature Research Reporting Summary linked to this article.

## Supplementary information

Supplementary Information

Peer Review File

Reporting Summary

## Data Availability

The PGZL1 HC and LC variable region sequences have been deposited into Genbank, accession MK497833–MK497838. The atomic coordinates and structure factors of PGZL1 variants have been deposited in the Protein Data Bank, with accession codes: 6O3D (PGZL1); 6O3G (PGZL1-MPER_671-683_); 6O3J (PGZL1-MPER_671-683_-06:0 PA); 6O3K (H4K3); 6O3L (H4K3-MPER_671-683_); 6O3U (H4K3-06:0 PA); 6O41 (PGZL1 gVmDmJ-Protein G); and 6O42 (PGZL1 gVmDmJ-MPER_671-683_-06:0 PA). The cryo-EM reconstruction of full-length AMC011-PGT151-PGZL1 complex has been deposited in the Electron Microscopy Data Bank with accession code EMD-0620. Env sequences and browser-based visualizations are available at https://flea.ki.murrell.group/view/PG13/sequences/. Datasets generated during and/or analyzed during the current study are included in the supplementary Source Data file.

## Source data

Source Data

## References

[1] Burton DR, Hangartner L (2016). Broadly neutralizing antibodies to HIV and their role in vaccine design. Annu. Rev. Immunol..

[2] Kwong PD, Mascola JR (2018). HIV-1 vaccines based on antibody identification, B cell ontogeny, and epitope structure. Immunity.

[3] Molinos-Albert LM, Clotet B, Blanco J, Carrillo J (2017). Immunologic insights on the membrane proximal external region: a major human immunodeficiency virus type-1 vaccine target. Front. Immunol..

[4] Haynes BF, Moody MA, Verkoczy L, Kelsoe G, Alam SM (2005). Antibody polyspecificity and neutralization of HIV-1: a hypothesis. Hum. Antibodies.

[5] Krebs SJ (2019). Longitudinal analysis reveals early development of three MPER-directed neutralizing antibody lineages from an HIV-1-infected individual. Immunity.

[6] Irimia A, Sarkar A, Stanfield RL, Wilson IA (2016). Crystallographic identification of lipid as an integral component of the epitope of HIV broadly neutralizing antibody 4E10. Immunity.

[7] Ivanov II (2005). Development of the expressed Ig CDR-H3 repertoire is marked by focusing of constraints in length, amino acid use, and charge that are first established in early B cell progenitors. J. Immunol..

[8] Haynes BF (2005). Cardiolipin polyspecific autoreactivity in two broadly neutralizing HIV-1 antibodies. Science.

[9] Doyle-Cooper C (2013). Immune tolerance negatively regulates B cells in knock-in mice expressing broadly neutralizing HIV antibody 4E10. J. Immunol..

[10] Huang J (2012). Broad and potent neutralization of HIV-1 by a gp41-specific human antibody. Nature.

[11] Williams LD (2017). Potent and broad HIV-neutralizing antibodies in memory B cells and plasma. Sci. Immunol..

[12] Sanders RW (2013). A next-generation cleaved, soluble HIV-1 Env trimer, BG505 SOSIP.664 gp140, expresses multiple epitopes for broadly neutralizing but not non-neutralizing antibodies. PLoS Pathog..

[13] Cardoso RM (2005). Broadly neutralizing anti-HIV antibody 4E10 recognizes a helical conformation of a highly conserved fusion-associated motif in gp41. Immunity.

[14] Lee JH, Ozorowski G, Ward AB (2016). Cryo-EM structure of a native, fully glycosylated, cleaved HIV-1 envelope trimer. Science.

[15] Jardine JG (2016). HIV-1 broadly neutralizing antibody precursor B cells revealed by germline-targeting immunogen. Science.

[16] Andrabi R (2015). Identification of common features in prototype broadly neutralizing antibodies to HIV envelope V2 Apex to facilitate vaccine design. Immunity.

[17] Liao HX (2013). Co-evolution of a broadly neutralizing HIV-1 antibody and founder virus. Nature.

[18] Klein F (2013). Somatic mutations of the immunoglobulin framework are generally required for broad and potent HIV-1 neutralization. Cell.

[19] Soto C (2016). Developmental pathway of the MPER-directed HIV-1-neutralizing antibody 10E8. PLoS ONE.

[20] Simek MD (2009). Human immunodeficiency virus type 1 elite neutralizers: individuals with broad and potent neutralizing activity identified by using a high-throughput neutralization assay together with an analytical selection algorithm. J. Virol..

[21] He L. et al. Hidden lineage complexity of glycan-dependent HIV-1 broadly neutralizing antibodies uncovered by digital panning and native-like gp140 trimer. *Front. Immunol.* **8**, 1025 (2017). 10.3389/fimmu.2017.01025 PMC5573810 28883821

[22] Kong L. et al. Key gp120 glycans pose roadblocks to the rapid development of VRC01-class antibodies in an HIV-1-infected chinese donor. *Immunity* **44**, 939–950 (2016). 10.1016/j.immuni.2016.03.006 PMC4862659 27067056

[23] Hessell AJ (2010). Broadly neutralizing monoclonal antibodies 2F5 and 4E10 directed against the human immunodeficiency virus type 1 gp41 membrane-proximal external region protect against mucosal challenge by simian-human immunodeficiency virus SHIVBa-L. J. Virol..

[24] Finton KA (2014). Ontogeny of recognition specificity and functionality for the broadly neutralizing anti-HIV antibody 4E10. PLoS Pathog..

[25] Stano A (2017). Dense array of spikes on HIV-1 virion particles. J. Virol..

[26] Ma BJ (2011). Envelope deglycosylation enhances antigenicity of HIV-1 gp41 epitopes for both broad neutralizing antibodies and their unmutated ancestor antibodies. PLoS Pathog..

[27] Leaman DP, Zwick MB (2013). Increased functional stability and homogeneity of viral envelope spikes through directed evolution. PLoS Pathog..

[28] Rujas E (2017). Functional contacts between MPER and the anti-HIV-1 broadly neutralizing antibody 4E10 extend into the core of the membrane. J. Mol. Biol..

[29] Nakamura KJ (2010). 4E10-resistant HIV-1 isolated from four subjects with rare membrane-proximal external region polymorphisms. PLoS ONE.

[30] Gray ES (2008). 4E10-resistant variants in a human immunodeficiency virus type 1 subtype C-infected individual with an anti-membrane-proximal external region-neutralizing antibody response. J. Virol..

[31] Kim AS, Leaman DP, Zwick MB (2014). Antibody to gp41 MPER alters functional properties of HIV-1 Env without complete neutralization. PLoS Pathog..

[32] Zwick M. B. et al. Anti-human immunodeficiency virus type 1 (HIV-1) antibodies 2F5 and 4E10 require surprisingly few crucial residues in the membrane-proximal external region of glycoprotein gp41 to neutralize HIV-1. *J. Virol.* **79**, 1252–1261 (2005). 10.1128/JVI.79.2.1252-1261.2005 PMC538539 15613352

[33] Irimia A (2017). Lipid interactions and angle of approach to the HIV-1 viral membrane of broadly neutralizing antibody 10E8: Insights for vaccine and therapeutic design. PLoS Pathog..

[34] Brooks BR (2009). CHARMM: the biomolecular simulation program. J. Comput. Chem..

[35] van Gils MJ (2016). An HIV-1 antibody from an elite neutralizer implicates the fusion peptide as a site of vulnerability. Nat. Microbiol..

[36] Yuan C (2017). The number of overlapping AID hotspots in hermline IGHV genes is inversely correlated with mutation frequency in chronic lymphocytic leukemia. PLoS ONE.

[37] Charles ED (2013). Somatic hypermutations confer rheumatoid factor activity in hepatitis C virus-associated mixed cryoglobulinemia. Arthritis Rheum..

[38] Lang S (2017). Antibody 27F3 broadly targets influenza A group 1 and 2 hemagglutinins through a further variation in VH1-69 antibody orientation on the HA stem. Cell Rep..

[39] Kong L (2013). Hepatitis C virus E2 envelope glycoprotein core structure. Science.

[40] Correia BE (2010). Computational design of epitope-scaffolds allows induction of antibodies specific for a poorly immunogenic HIV vaccine epitope. Structure.

[41] Avnir Y (2017). Structural determination of the broadly reactive anti-IGHV1-69 anti-idiotypic antibody G6 and its idiotope. Cell Rep..

[42] Ingale S, Gach JS, Zwick MB, Dawson PE (2010). Synthesis and analysis of the membrane proximal external region epitopes of HIV-1. J. Pept. Sci..

[43] Cao L (2018). Differential processing of HIV envelope glycans on the virus and soluble recombinant trimer. Nat. Commun..

[44] Sarkar A (2018). Structure of a cleavage-independent HIV Env recapitulates the glycoprotein architecture of the native cleaved trimer. Nat. Commun..

[45] Torrents de la Peña A., et al. Similarities and differences between native HIV-1 envelope glycoprotein trimers and stabilized soluble trimer mimetics. *PLoS Pathog.* **15**, e1007920 (2019). 10.1371/journal.ppat.1007920 PMC6658011 31306470

[46] Rujas E (2018). Functional optimization of broadly neutralizing HIV-1 antibody 10E8 by promotion of membrane interactions. J. Virol..

[47] Kwon YD (2018). Surface-matrix screening identifies semi-specific interactions that improve potency of a near pan-reactive HIV-1-neutralizing antibody. Cell Rep..

[48] Gray E. S. et al. Broad neutralization of human immunodeficiency virus type 1 mediated by plasma antibodies against the gp41 membrane proximal external region. *J. Virol.* **83**, 11265–11274 (2009). 10.1128/JVI.01359-09 PMC2772769 19692477

[49] Landais E (2017). HIV envelope glycoform heterogeneity and localized diversity govern the initiation and maturation of a V2 apex broadly neutralizing antibody lineage. Immunity.

[50] He L (2014). Toward a more accurate view of human B-cell repertoire by next-generation sequencing, unbiased repertoire capture and single-molecule barcoding. Sci. Rep..

[51] Otwinowski Z, Minor W (1997). Processing of X-ray diffraction data collected in oscillation mode. Methods Enzymol..

[52] Kabsch W (2010). XDS. Acta Crystallogr. D Biol. Crystallogr..

[53] McCoy AJ (2007). Phaser crystallographic software. J. Appl. Crystallogr..

[54] Emsley P, Lohkamp B, Scott WG, Cowtan K (2010). Features and development of Coot. Acta Crystallogr. D Biol. Crystallogr..

[55] Adams, P. D. et al. PHENIX: a comprehensive Python-based system for macromolecular structure solution. *Acta Crystallogr. D Biol. Crystallogr.* **66**, 213–221 2010. 10.1107/S0907444909052925 PMC2815670 20124702

[56] Lorizate M (2013). Comparative lipidomics analysis of HIV-1 particles and their producer cell membrane in different cell lines. Cell Microbiol..

[57] Rantalainen K (2018). Co-evolution of HIV envelope and Apex-targeting neutralizing antibody lineage provides benchmarks for vaccine design. Cell Rep..

[58] Potter CS (1999). Leginon: a system for fully automated acquisition of 1000 electron micrographs a day. Ultramicroscopy.

[59] Zheng SQ (2017). MotionCor2: anisotropic correction of beam-induced motion for improved cryo-electron microscopy. Nat. Methods.

[60] Zhang K (2016). Gctf: Real-time CTF determination and correction. J. Struct. Biol..

[61] Voss NR, Yoshioka CK, Radermacher M, Potter CS, Carragher B (2009). DoG Picker and TiltPicker: software tools to facilitate particle selection in single particle electron microscopy. J. Struct. Biol..

[62] Punjani A, Rubinstein JL, Fleet DJ, Brubaker MA (2017). cryoSPARC: algorithms for rapid unsupervised cryo-EM structure determination. Nat. Methods.

[63] Zivanov J (2018). New tools for automated high-resolution cryo-EM structure determination in RELION-3. eLife.

[64] Kumar V. et al. Long-read amplicon denoising. *Nucleic Acids Res*., Aug 16. pii: gkz657. doi: 610.1093/nar/gkz1657 (2019). 10.1093/nar/gkz657 PMC6765106 31418021

[65] Katoh K, Standley DM (2013). MAFFT multiple sequence alignment software version 7: improvements in performance and usability. Mol. Biol. Evol..

[66] Lengauer T, Sander O, Sierra S, Thielen A, Kaiser R (2007). Bioinformatics prediction of HIV coreceptor usage. Nat. Biotechnol..

