# Supplementary material for: An MPER antibody neutralizes HIV-1 using germline features shared among donors
Source: Nat Commun. 2019 Nov 26;10:5389. doi: 10.1038/s41467-019-12973-1 (PMC6879610; doi:10.1038/s41467-019-12973-1)
Supplement: Supplementary file 1 — Supplementary Information [file 41467_2019_12973_MOESM1_ESM.pdf]

Supplementary Information

An MPER Antibody Neutralizes HIV-1 Using Germline Features Shared Among Donors

Zhang *et al.*

List of Content

Supplementary Figures 1-9

Supplementary Tables 1-8

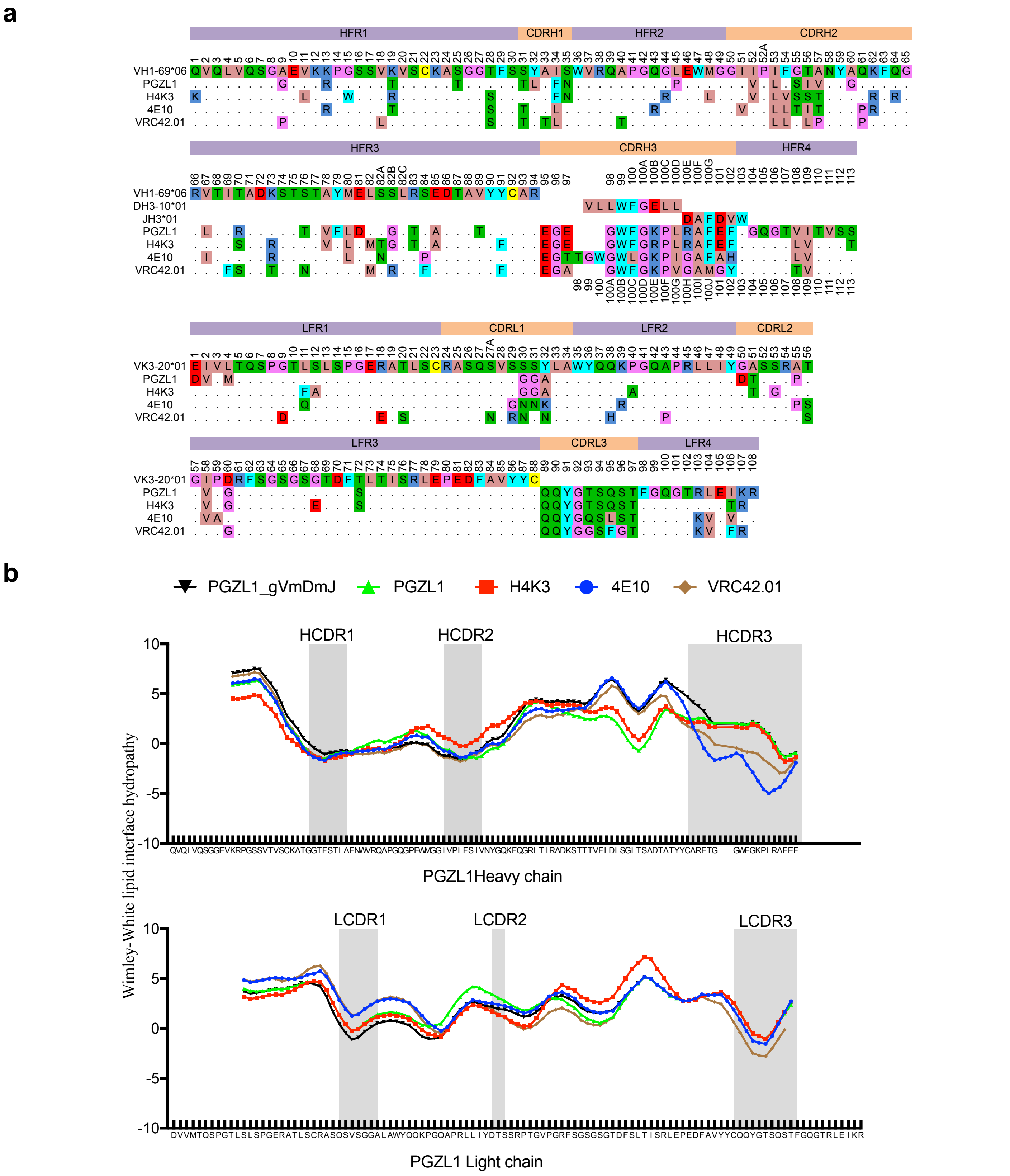

Supplementary Figure 1. Amino-acid sequence and calculated lipid insertion propensity of MPER bnAbs. **a** Amino-acid alignment of PGZL1, H4K3, 4E10 and VRC42.01. Amino acid residues are colored by their physico-chemical properties: pink, aliphatic; orange, aromatic; magenta, Gly and Pro; yellow, Cys; green, hydrophilic; red, acidic; blue, basic. The CDRs are indicated according to Kabat. **b** Lipid insertion propensity plots of the five antibodies for the heavy chains (top panel) and light chains (bottom panel). Lipid insertion property was calculated using the Wimley-White lipid interface hydropathy scale as computed with MPEx software; curve smoothing utilized the default window size of 19 amino acids. Source data for panel (**b**) is provided as a Source Data file.

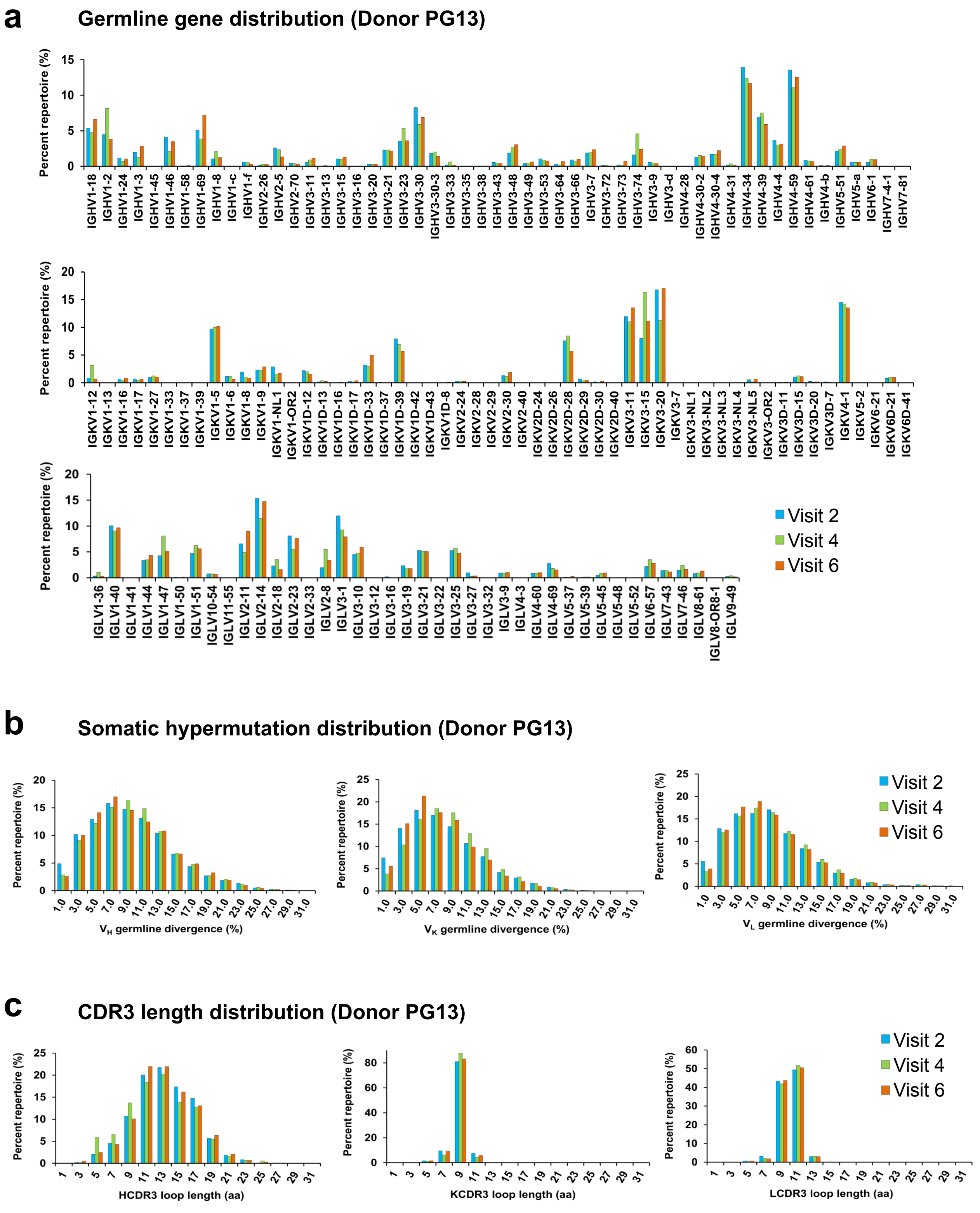

Supplementary Figure 2. Unbiased B cell repertoire profiles of donor PG13 at three time points. Distributions are plotted. **a** Germline V gene usage for heavy and light (κ and λ) chains. **b** Germline gene divergence, or extent of SHM. **c** CDR3 loop length (H, heavy chain; K, κ chain; L, λ chain). Color coding denotes the time point analyzed, with sample visits V2 shown in blue, V4 in green, and V6 in orange.

a

Visit	CDR3 identity >80% (HC), >85% (KC)	Translatable to amino acid sequences	Non-redundant sequences	Clustering based on a SeqID of 90%	SeqID-based selection of representative sequences
#2	727 HCs	555 HCs	366 HCs	65 HC clusters	10 HC representatives
	93 KCs	23 KCs	22 KCs	10 KC clusters	4 KC representatives
#4	759 HCs	597 HCs	377 HCs	48 HC clusters	8 HC representatives
	112 KCs	28 KCs	28 KCs	12 KC clusters	3 KC representatives
#6	853 HCs	644 HCs	460 HCs	79 HC clusters	9 HC representatives
	141 KCs	14 KCs	14 KCs	8 KC clusters	3 KC representatives

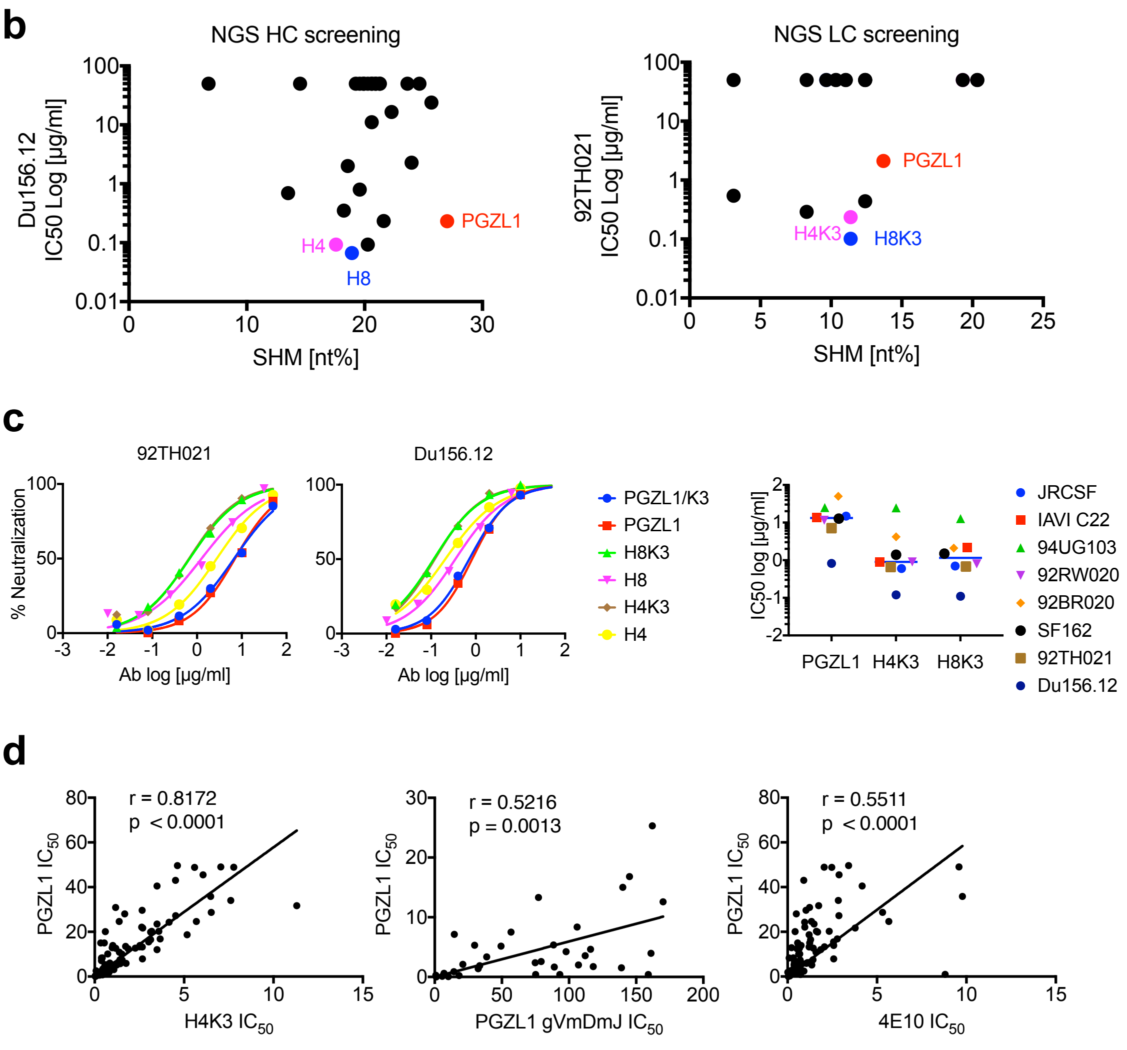

Supplementary Figure 3. Neutralization of HIV-1 by PGZL1 recombinant antibodies selected from NGS lineage analysis of the Donor PG13 antibody repertoire. **a** Details of bioinformatics procedure used for selecting PGZL1-like sequences. **b** Scatter plots showing neutralization (IC_{50}) of HIV-1 isolate Du156.12 vs SHM (nucleotide level, nt%) with heavy chains (HC) that were initially paired with the wildtype PGZL1 light chain (left panel). From this screen, the heavy chains that yielded the highest neutralization potency, H4 and H8, were subsequently paired with a panel of light chains (LC) and subsequently assayed for neutralization of HIV-1 isolate 92TH021 (right panel). **c** Neutralization curves of 92TH021 and Du156.12 by PGZL1 and NGS recombinant variants are shown on the left and middle panels, respectively. On the right panel, log IC_{50}s are plotted for each antibody against the 6-isolate cross-clade panel. **d** Correlation of neutralization (IC_{50}s) against a 130-member panel of HIV isolates comparing PGZL1 and H4K3 (left panel, n=89, r=0.8172, p<0.0001), PGZL1 and PGZL1 gVmDmJ (middle panel, n=35, r=0.5216, p=0.0013), as well as PGZL1 and 4E10 (right panel, n=89, r=0.5511, p<0.0001). Source data for panels (**b**), (**c**), (**d**) are provided as a Source Data file.

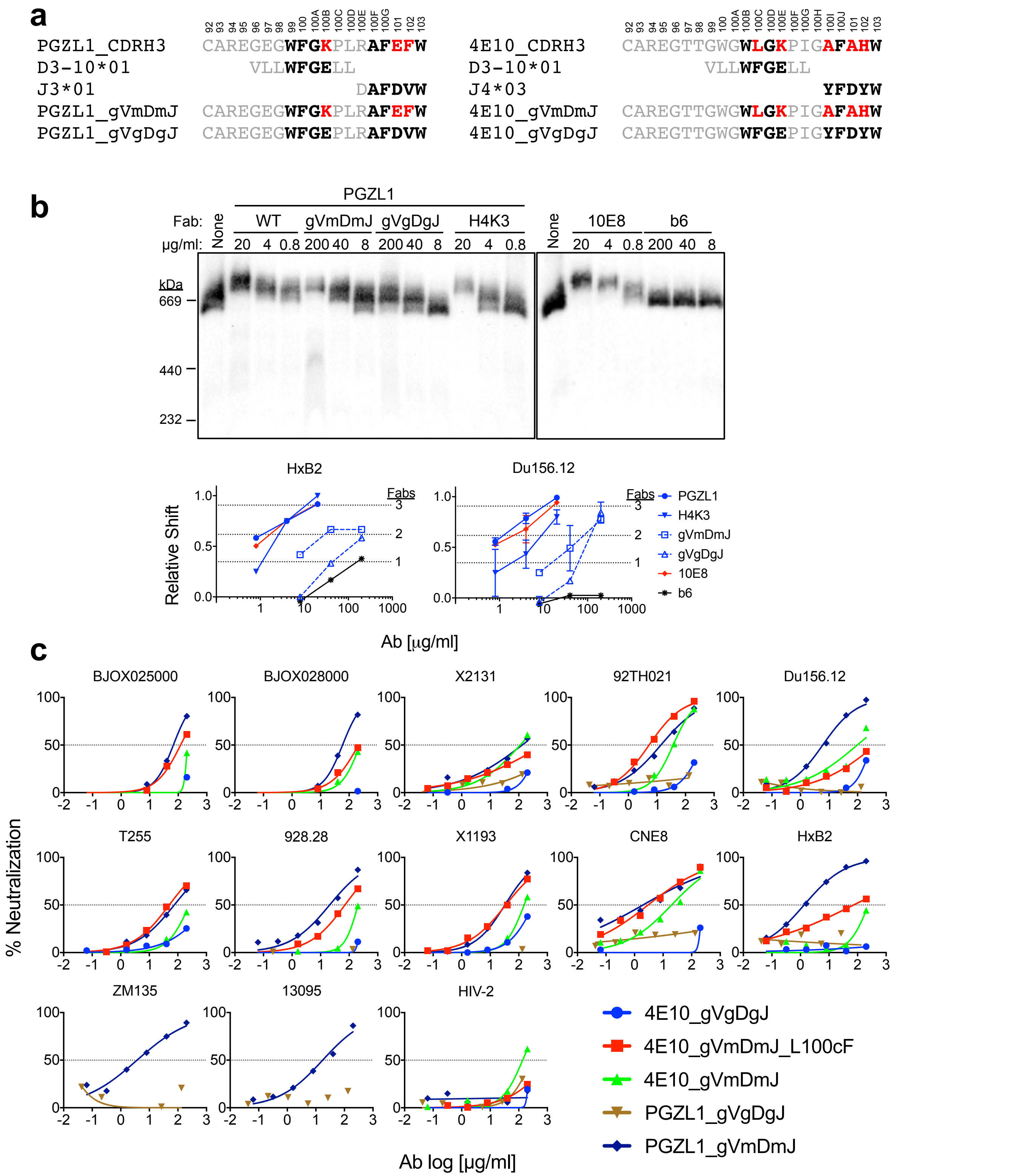

Supplementary Figure 4. CDRH3 sequences, HIV Env binding characteristics, and HIV neutralization of inferred germline precursors of PGZL1 and 4E10. **a** Inferred CDRH3 germline sequences were aligned to those of PGZL1 and 4E10. Residues conserved from germline are in bold black, and SHMs are in red. **b** BN-PAGE Env mobility shift assay. BN-PAGE Western blot analysis of HIV Env ADA-CM and relative gel mobility shift in the presence of PGZL1 variant antibodies was performed as in Fig. 2. Gel mobility shift data were also acquired for HIV-1 HxB2 and Du156.12. Relative gel mobility shifts were calculated as described in Fig. 2 for ADA-CM. The error bars represent the standard deviation of n=2 biologically independent experiments. **c** Neutralization of indicated PGZL1 and 4E10 inferred germline antibodies against 13 HIV pseudoviruses in the TZM-bl assay. Source data for panels (**b**), (**c**) are provided as a Source Data file.

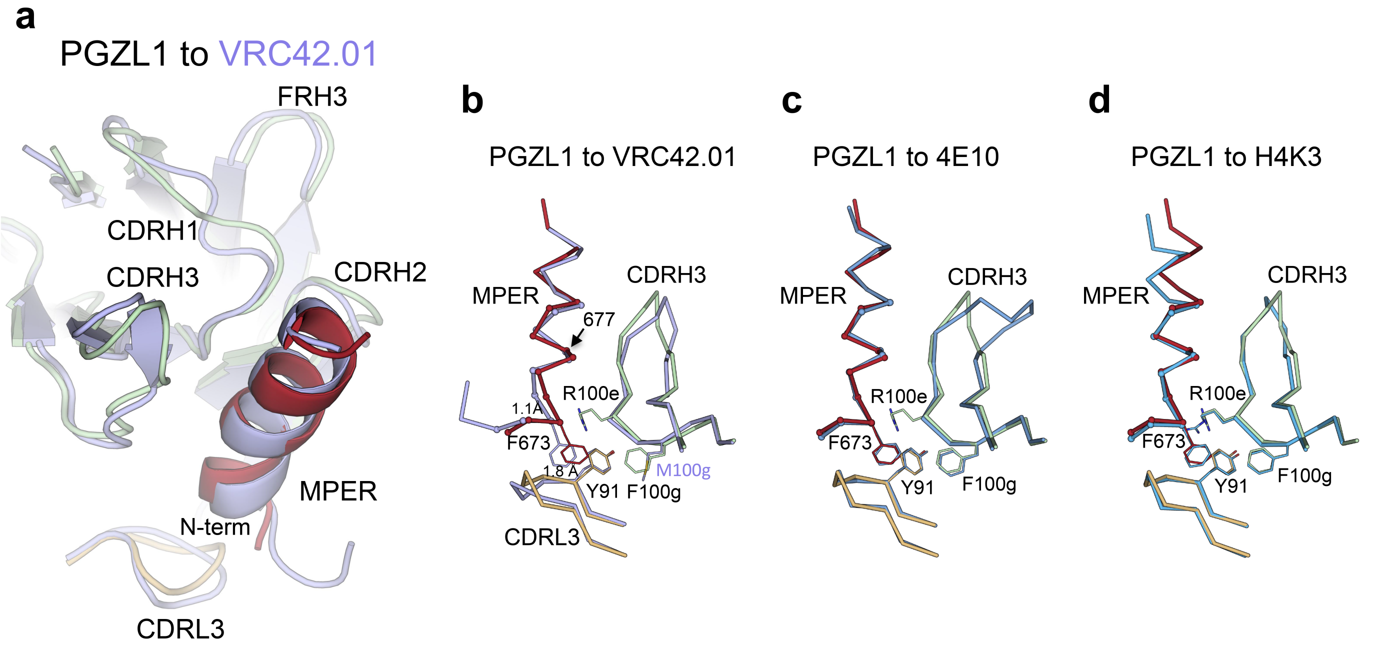

Supplementary Figure 5. PGZL1 and VRC42.01 structure comparison. **a** Superposition of variable domain of PGZL1 (green-HC; yellow-LC; red-MPER) to VRC42.01 (blue; PDB 6MTO) reveals slight differences in the position of the MPER N-term region. **b** Zoom into the superposition of PGZL1 (green-HC; yellow-LC; red-MPER) to VRC42.01 (blue) highlighting aromatic residues (sticks) in the combining site and N-term region of the epitope. Cα atoms of F_{673} in the two structures are 1.1 Å apart and equivalent atoms of their aromatic rings are up to 1.8 Å apart. The position of F_{100g} in PGZL1 corresponds to M_{100g} in VRC42.01. Residue 677 is an asparagine in our MPER peptide and a lysine in the MPER in the VRC42.01 structure and is indicated with an arrow. **c** Zoom into the superposition of PGZL1 (green-HC; yellow-LC; red-MPER) to 4E10 (blue; PDB 2FX7) shows similar positions of the MPER epitope and Fab residues (sticks) in the two structures. **d** Zoom into the superposition of PGZL1 (green-HC; yellow-LC; red-MPER) to H4K3 (blue) shows similar positions of the MPER epitope and Fab residues (sticks) in the two structures. Cα atoms of the MPER epitope residues interacting with each antibody are shown as small spheres in panels (**b**), (**c**), (**d**).

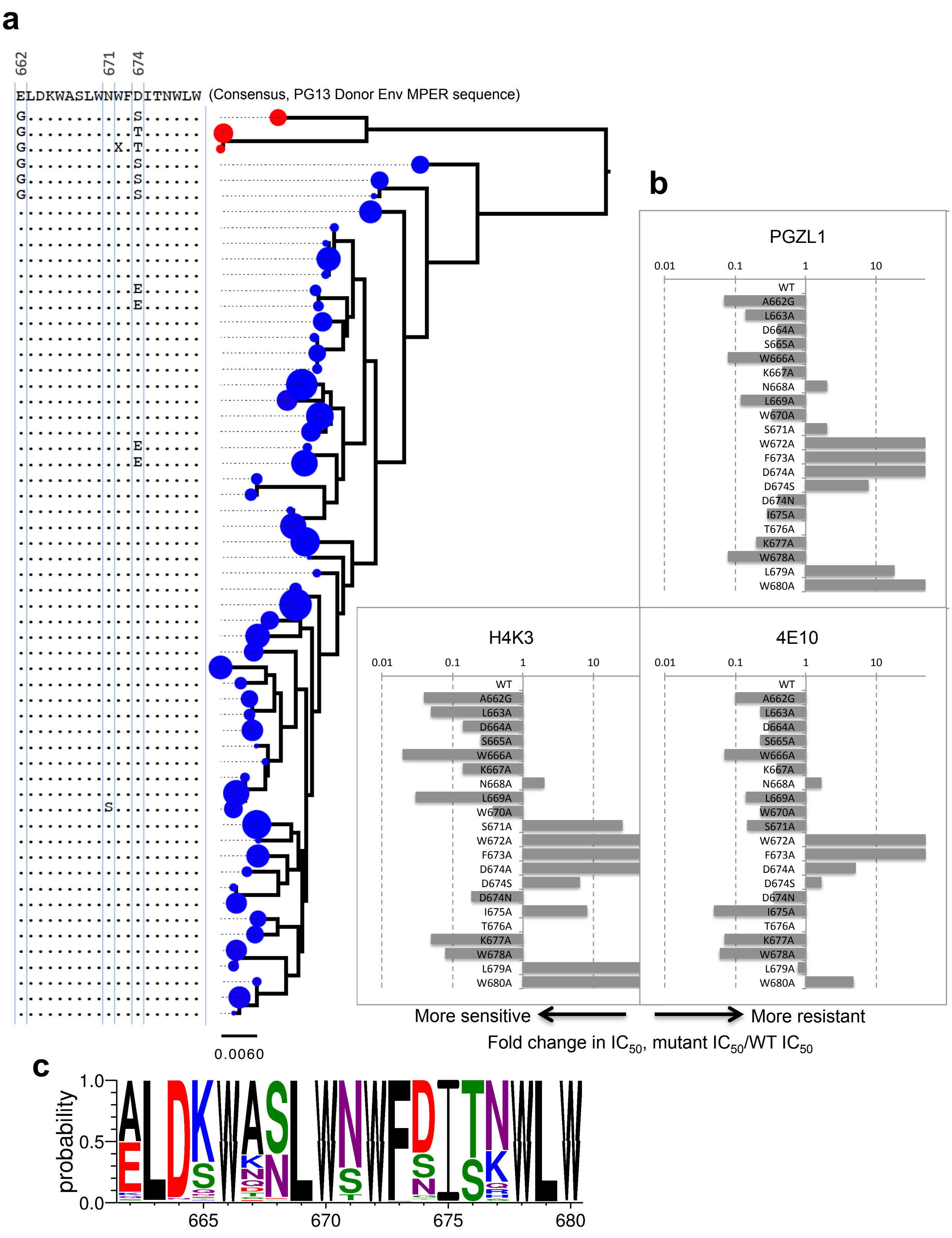

Supplementary Figure 6. Isolation and phylogenetic analysis of PG13 donor Envs and sensitivity of HIV COT6 MPER mutants to neutralization by PGZL1 antibodies. **a** Alignment of MPER sequences obtained from Envs rescued from plasma-derived viral RNA of donor PG13 using long-read NGS. An X in the MPER sequence represents a stop codon. The adjacent phylogeny is annotated with circles whose size corresponds to the number of variant frequency and whose color indicates whether the Env is predicted to be CXCR4-tropic (red) or CCR5-tropic (blue). The units of the scale bar are nucleotide substitutions per site. **b** Relative neutralization sensitivity of HIV COT6.15 wildtype vs COT6.15 MPER mutants to PGZL1, H4K3 and 4E10 antibodies. Results are reported as fold-increase in IC_{50} relative to wildtype COT6.15. **c** Logoplot of MPER sequence for all Envs in the LANL database. Source data for panel (**b**) is provided as a Source Data file.

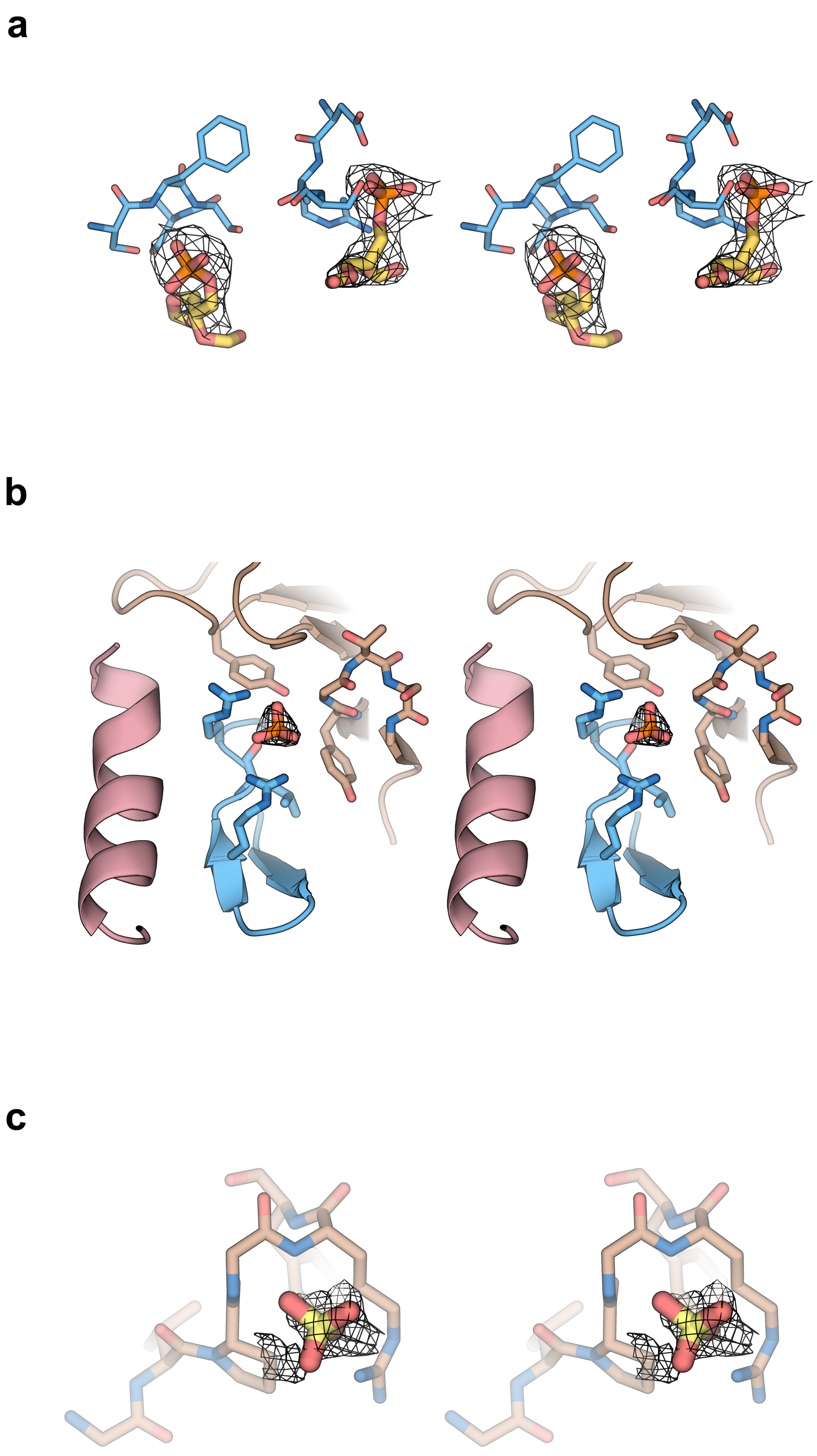

Supplementary Figure 7. Stereo images of the electron density maps illustrating binding of the ligands**. a** Stereo image of the initial Fo-Fc map (3σ level; black mesh) for two 06:0 PA (yellow sticks) bound to H4K3 residues (blue sticks). **b** Stereo image of the initial Fo-Fc map (3σ level; black mesh) corresponding to a bound PO4 (orange). H4K3 residues are shown as sticks (blue - HC and brown – LC) and MPER$_{671\text{-}683}$ as pink helix. **c** Stereo image of the initial Fo-Fc map (3σ level; black mesh) corresponding to a bound SO4 (yellow) surrounded by H4K3 residues (brown sticks).

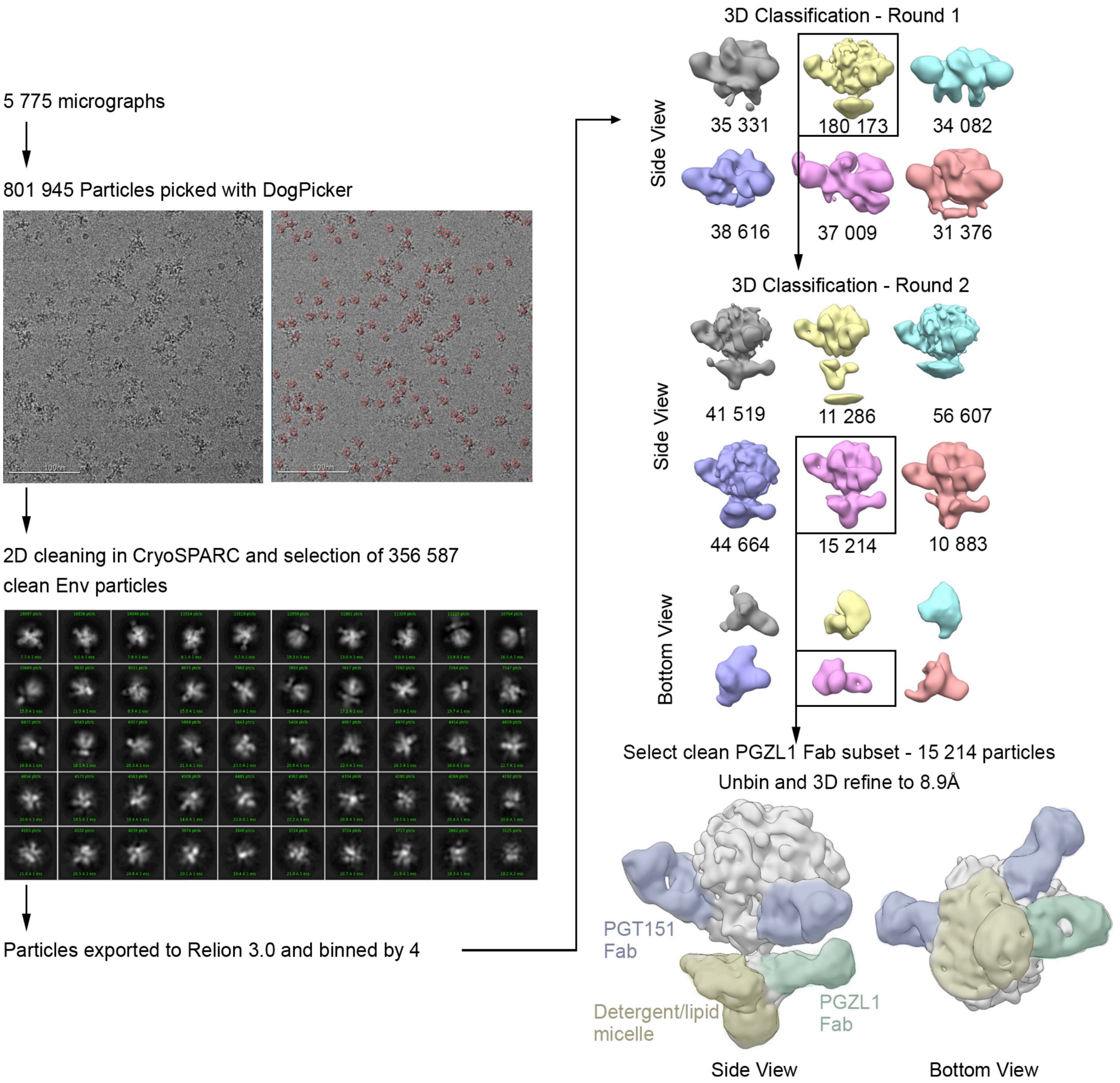

Supplementary Figure 8. Cryo-EM data processing workflow for full-length AMC011 Env with PGZL1 and PGT151 Fabs. Particles were picked with DoGPicker and after 2D classification in cryoSPARC, clean particle stack was further curated with two rounds of 3D classification, yielding a final class of 15214 particles with one PGZL1 Fab and two PGT151 Fabs per Env trimer and refined to 8.9 Å resolution.

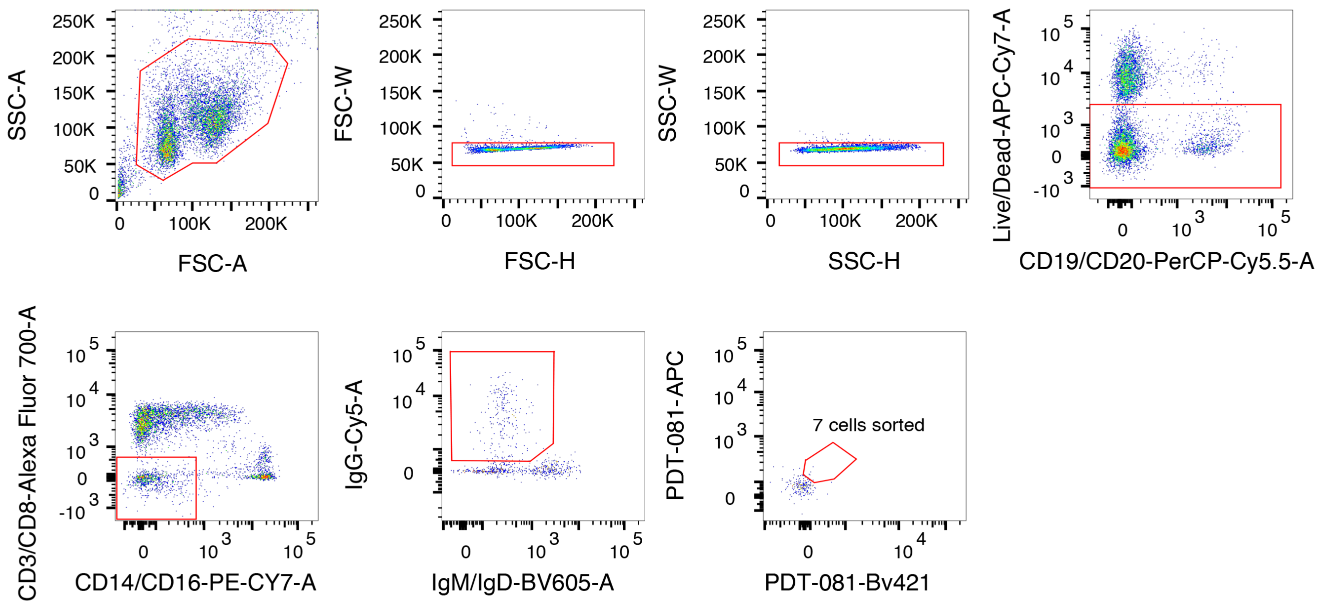

Supplementary Figure 9. FACS strategy for isolating single MPER-specific B cells.

Supplementary Table 1. Neutralization of HIV-1 and HIV-2 (HIV-1 MPER) chimera viruses by PG13 donor serum and mAbs

Virus	MPER	PG13 Plasma (±competitor)[a]		Monoclonal Ab (IC_{50} µg/ml)		
		-	+	PGZL1	H4K3	4E10
HIV-2 C1	ELLALDKWASLWNWFDITKWLWYIK	6400	940	0.98	n.d.	0.80
HIV-2 C3	ELLALDKWASLWNWFDLASWVKYIQ	<80	n.d.	>10	>10	>10
HIV-2 C4	ELQKLNSWDVFGNWFDITKWLWYIK	6400	n.d.	2.38	1.17	1.41
HIV-2	ELQKLNSWDVFGNWFDLASWVKYIQ	<80	n.d.	>10	>10	>10
HIV-1 Du156.12	DLLALDRWQNLWNWFDITNWLWYIK	1140	280	0.623	0.270	0.101
HIV-1 Du156.12[b]	DLLALDRWQNLWNWFDITNWLWYIK	1140	1911	0.623	0.270	0.101

[a]ID_{50} in reciprocal plasma dilution against each virus in the presence (+) or absence (-) of 10 µg/ml MPER peptide (residues 654-683). n.d., not determined.
[b]The fusion peptide (residues 512-534) was used as competitor.

Supplementary Table 2. Germline gene usage and characteristics of HIV-1 MPER bnAbs

Abs	Putative heavy chain gene alleles			V (nt%) mutation frequency	HCDR3 aa sequence	HCDR3 length (aa)	Isotype
PGZL1	IGHV1-69*06	IGHD3-10*01	IGHJ3*01	20.9	EGEGWFGKPLRAFEF	15	IgG1
4E10	IGHV1-69*10	IGHD3-10*01	IGHJ4*03	6.8	EGTTGWGWLGKPIGAFAH	18	IgG3
VRC42.01	IGHV1-69*10	IGHD3-10*01	IGHJ4*03	10.8	EGAGWFGKPVGAMGY	15	IgG1
10E8	IGHV3-15*05	IGHD3-3*01	IGHJ1*01	21.5	TGKYYDFWSGYPPGEEYFQD	20	IgG3
DH511.2	IGHV3-15*01	IGHD3-3*01	IGHJ6*03	17.6	TMDEGTPVTRFLEWGYFYYYMAV	23	IgG3
DH517	IGHV4-34*01	IGHD3-16*01	IGHJ6*01	18.1	ARGTGVVVGGSWTVPPGMAYYLDV	24	IgG3
Z13	IGHV4-59*03	IGHD2-15*01	IGHJ6*03	17.7	VAIGVSGFLNYYYYMDV	17	N.D.
2F5	IGHV2-5*02	IGHD3-3*01	IGHJ6*02	12.1	RRGPTTSSGVPIARGPVNAMDV	22	IgG3
Abs	Putative light chain gene alleles			V (nt%) mutation frequency	LCDR3 aa sequence	LCDR3 length (aa)	
PGZL1	IGKV3-20*01		IGKJ5*01	12.6	QQYGTSQST	9	
4E10	IGKV3-20*01		IGKJ2*01	4.7	QQYGQSLST	9	
VRC42.01	IGKV3-20*01		IGKJ1*01	5.7	QQYGGSFGT	9	
10E8	IGLV3-19*01		IGLJ3*02	15.2	SSRDKSGSRLSV	12	
DH511.2	IGLK1-39*01		IGKJ2*03	15.7	QENYNTIPSLS	11	
DH517	IGLV3-19*01		IGLJ2*01	13.4	ASRDRSGDRLGV	12	
Z13	IGKV3-11*01		IGKJ1*01	6.0	QQRSDWPRT	9	
2F5	IGKV1D-13*02		IGKJ4*01	11.8	QQLHFYPHT	9	

N.D. Not determined

Supplementary Table 3. Unbiased antibody repertoire analysis of HIV-1-infected patient PG13 from Protocol G cohort[a].

Visit	N_{read}	N_{assign}	Chain	N_{chain}	<length>(nt)	$Perc_{usable}$ (%)
#2 (18-18-2008)	5,692,576	4,161,595	H	2,026,540	554.2	58.2%
			κ	1,095,554	563.4	53.9%
			λ	1,039,501	581.8	56.7%
#4 (03-12-2009)	4,412,667	3,486,756	H	1,793,460	583.4	63.9%
			κ	882,793	570.1	58.5%
			λ	810,503	588.9	62.2%
#6 (05-18-2009)	5,040,531	3,934,837	H	1,715,839	583.8	66.6%
			κ	1,178,148	568.1	59.4%
			λ	1,040,850	589.0	60.4%

[a] The unbiased antibody repertoire analysis was performed using a human 5'-RACE PCR procedure for library preparation on the Ion Chef instrument and long-read sequencing on the Ion GeneStudio S5 NGS platform. Listed items include the time point of the patient visit, total number of raw reads, number of remaining reads after germline gene assignment with a cutoff E-value of 10^{-3}, antibody chain type (H, κ, and λ), number of antibody chains, average read length, and total number of usable sequences after Antibodyomics 1.0 pipeline processing and bioinformatics filtering with a V-gene alignment cutoff of 250 bp. Nine antibody chain libraries were barcoded according to three time points and pooled on one Ion 530 chip for the NGS experiment.

Supplementary Table 4. PGZL1-like heavy chain and light chain sequences selected from NGS lineage analysis.

Type	Name	NGS Idx	SHM (nt %)	Sequence identity (nt %)	Amino acid sequence of variable domain
HC	V2H1	4140766	6.757	80.6	QVQLVQSGAEVKKPGSSVKVSCKASGGTFSNDVISWVRQAPGQGLEWMGRVIPILDITNYAQKFQGRVTITADKSTSTVYMDLSSLRSEDTAVYFCAREGEGWFGKPLRAFEVWGQGTQITVSS
HC	V2H2	3634260	13.514	79.3	QVQLVQSGAEVKKPGSSVKVSCKASGSTFSSYAISWVRQAPGQGLEWVGGIVPLVSSTNYAQRFRGRVTISADRSTSTVYLEMTGLTSADTAVYFCAREGEGWFGRPLRAFEFWGQGTLVTVST
HC	V2H3	1505330	19.257	76.1	KVQLVQSGAELKKPWSSVRVSCKASGGSFSSYAFNWVRQAPGQKLEWLGGIASLLVSRPSYAQRFRGRITISADRSATTVYLEMTGLTSADTAVYFCAREGEGWFGKPLRAFEFWGQGTLVTVST
HC	V2H4	1592240	17.568	77.2	KVQLVQSGAELKKPWSSVRVSCKASGGSFSSYAFNWVRQAPGQRLEWLGGIVPLVSSTNYAQRFRGRVTISADRSTSTVYLEMTGLTSADTAVYFCAREGEGWFGRPLRAFEFWGQGTLVTVST
HC	V2H5	2499141	18.581	92.2	GVQLVQSGAEVKKPGSSMTVSCKATGGTFSSLAFNWVRQAPGQGPEWMGGICPVFSALVNYGQRFQGRLTIRADKSTTTVYLDLIRLTSDDTATYYCAREGEGRFGKPLRAFEVWGQGTQITVSS
HC	V2H6	2684075	20.608	75.0	KVQLVQSGAEVKRPGSSVTISCKDRGGSFSSYAFNWVRQAPGQGLEWMGGIIPLISIANYASRRFRGRVTITADRSTSSIFLDLTRLTSVDTALYFCAREGEGWFGKPLGAFEFWGQGTAVTVTS
HC	V2H7	133943	21.622	95.2	GVQLVQSGAEVKRPGSSVTVSCKATGGTFSTLAFSWVRQAPGQGPEWMGGIVPLFTIVNYGQRFQGRLTIRADKSTTTVFLDLSGLTSADTATYYCAREGEGWFGKPLRALEIWGQGTVITVSS
HC	V2H8	1537468	18.919	76.9	RVQLVQSGAELKKPWSSVRVSCKASGGSFSSYAFSWVRQAPGQGLEWLGGIVPLVSSTNYAPKFRGRITISADRSANTVYLEMTRLTSADTAVYFCAREGEGWFGRPLRAFEFWGQGTLVTVST
HC	V2H9	891566	23.986	74.5	KVQLVQSGAEVMRPGSSGYLSCKASGGSFSSYAFNWVRQAPGQGLEWMGGIIPLISIANYAEEFRGRVTITADRSTSSIFLDLTRLTSVDTALYFCAREGEGWFGKPLGAFEFWGQGTAVTVTS
HC	V2H10	2745751	25.676	73.1	RVQLVQSGAEVKRPGSSVTIACKASGGSCSSYALHWERQARGQGLEWMGGIMPPYRVANYAEELRGRVTMTGDRSTSSIFLDLTRLTSVDTALYFCAREGEGWFGKPLGAFEFWGQGTAVTVTS
HC	V4H1	4221067	14.527	79.3	QVQLAQSGTEVKKPGSSVKVSCKSSGGTSSNYAITWVRQAPGQGLEWMGGIVPLVSSTNYAQRFKGRVTISADRSTSTVFMEVIRLTSEDTGVYFCAREGEGWFGKPLRAFEFWGHGTAVTVSS
HC	V4H2	2042752	20.608	95.2	GVQLVQSGAEVNEGPGSSVEVSCKATGGTFSTLAFNWVRQAPGQGPEWMGGIVPLFSIVNYGQRFQGRVTIRADKSTTTVFLDLSRLTSADTATYYCAREGEGWFGKPLRALEIWGQGTVITVSS
HC	V4H3	3465249	19.595	94.6	GVQLVQSGAEVKRPGSSMTVSCRATGGTFSSLAFNWVRQAPGQGPEWMGREIVPLFRIANYGQKFQGRLTIRADKSTTTIYLDLSSLTSADTATYYCAREGEGWFGKPLRAFEFWGQGTVITVSS
HC	V4H4	176346	18.243	78.0	KVQLVQSGAELKKPWSSVNEVSCKVSGGSFSSYAFNWVRQAPGQRLEWLGGIVPLVSSTNYAQRFRGRITISADRSTSTVYLEMTGLTSADTAVYFCAREGEGWFGKPLRAFEFWGQGTLVTVST
HC	V4H5	1254417	20.946	75.5	KVQLVQSGAEVKRPGSSVTISCKGTRGGSFSSYAFNWVRQAPGLGLEWMGGIIPLISIANYAERFRGRVTITADRSTSSIFLDLTRLTSVDTALYFCAREGEGWFGKPLGAFEFWGQGTAVTVTS
HC	V4H6	1122859	20.608	94.9	RVQLVQSGAEVKRPGSSVTVSCKATGGTFSTLAFSWVRQAPGQGPEWMGGIVPLFTIVNYGQRFQGRLTIRADKSTTTVFLDLSGLTSADTATYYCAREGEGWFGKPLRAPEIWGQGTVITVSS
HC	V4H7	1501103	20.27	89.0	GVQLVASGAEVKKPGSSVEVSCKATGGTFNSLAFNWVRQAPGQGPEYMGGIVPLFSIVNYGQRFQGRLTIRADKSTTTVYMDLNRLTSDDTATYYCAREGEGWFGKPLRAFQLWGQGTVITVSS
HC	V4H8	3307001	23.649	75.8	KVQLVQSGAEVKRPGSSVTISCKDSGGSFSSYAFNWVRQAPGQGLEWMGGIIPLISSTNYAEKFRGRVTITADRSTSSIFLDLTRLTSADTALYFCAREGEGWFGKPLGAFEFWGQGTAVTVTS
HC	V6H1	1008039	20.946	77.2	KVQLVQSGDVKLKTPWSSVRVSCKASGGSFSSYAFNWVRQAPGQRLEWLGGIVPLVSSTNYAQRFRGRVTISADRSANTVYLEMTGLTSADTAIYFCAREGEGWFGKPLRAFEFWGQGTLVSVST
HC	V6H2	1199275	19.932	76.9	KVQLVQSGAELKKPWSSVRVSCKASGGSFSSYAFNWVRQAPGQRLECMGGIVPLVSSTNYAQRFRGRITISADRSASTVYLEMTGLTSADTAVYFCAREGEGWFGKPLRAFEFWGQGTLVAVST
HC	V6H3	1330540	21.284	76.9	KVQLVQSGAELKKPWSSVRVSCKATGGSFSSYAFNWVRPAPGQRLEWLGGIVPLVSSTNYAQRFRGRITISADRSASTVYLEMTGLTSADTAVYFCAREGEGWFGRPLRAFEFWGQGTLVTVST
HC	V6H4	1944093	21.284	76.6	KVQLVQSGAEVKRPGSSVTISCKASGGSFSSYAFNWVRQAPGLGLEWMGGIIPLISIANYAQRRFRGRVTITADRSTSSIFLDLTRLTSVDTALYFCAREGEGWFGKPLGAFEFWGQGTAVTVTS
HC	V6H5	1151354	19.595	76.6	KVQLVQSGAELKKPWSSMRVSCKASGGSFSSYAFNWVRQAPGQRLEWLGGIVPLVSSTNYAQRFRGRITISADRSASTVYLEMTGLTSADTAVYFCAREGEGWFGKPLRAFEFWGQGTLVTVSA
HC	V6H6	774842	19.932	93.0	GVQLVQSGAEVKKPGSSVTVSCKATGGTFSSLAFNWVRPMAPGQGPEWMGGIVPLFSIVNYGQRFQGRLTIRADKSTTTVYLDLIRLTSDDTATYYCAREGEGWFGKPLRAFEFWGQGTLITVSS
HC	V6H7	1093434	20.270	89.8	GVQLVQSGAEVKKPGVSVTVSCKATGGTFSSLAFNWVRQAPGQGPEYMGGIVPLFSIVNYAQRFQGRLTIRADKSTTTVYMDLNRLTSDDTATYYCAREGEGWFGKPLRAFQLWGQGTVITVSS
HC	V6H8	2421650	22.297	95.2	GVQLVQSGAEVKRPGSSVEVSCKATGGTFSTLAFSWVRQAPGQGPEWMGGIVPLFTIVNYGQRFQGRLTIRADKSTTTVFLDLSGLTSADTATYYCAREGEGWFGKPLRALEIWGQGTVITVSS
HC	V6H9	3522135	24.662	73.4	KVQLVQSGAEMKRPGSSVHAACKDRGGSFSSYAIIWVRQARELGFEWMGGIIPLLSRANYAQRWFRGRVTITAHESTSSIFLDLTRLTSVDTALYFCAREGEGWFGKPLGAFEFWGQGTAVTVTS
KC	V2K1	2224128	8.276	85.2	EIVLTQSPGTLSLSPGERATLSCRASQSVNSNYLAWYQQKPGQAPRLLIYRALGRATGIPDRFSGSGSGTDFTLTISRLEPEDFAVYYCQQYGTSESTFGQGTRLEIR
KC	V2K2	5128161	11.034	92.6	EIVLTQSPGTLSLSPGERATLSCRASQSVSGGALAWYQQKAGQAPRLLIYDTSGRATGVPGRFSGSGSETDFSLTISRLEPEDFAVYYCQQYGTSQSTFGQGTRLETR
KC	V2K3	5476422	11.379	93.5	EIVLTQSPGTFALSPGERATLSCRASQSVSGGALAWYQQKAGQAPRLLIYGTSGRATGVPGRFSGSGSETDFSLTISRLEPEDFAVYYCQQYGTSQSTFGQGTRLETR
KC	V2K4	1507161	19.310	88.0	RIVLTQSPGTLSLSPGARATLSCRASQSVSGGSLAWYQQKAGRAPRSVIYDAVRRATAIPGRFSGSGSETDFSLTISRLEPEDLAVYYCQQYGTSQSTFGQGTRLEIR
KC	V4K1	1064505	12.414	92.0	EIVLTQPPGNFWSLSPGQRATLSCRAGQSVSGGSLAWYQQKAGQAPRLLIYDTSSRATGVRDRFSGSGSETDFSLTISRLEPEDFAVYYCQQYGTSQSTFGQGTRLEMR
KC	V4K2	2329152	12.414	92.0	EIVLTQSPVTLSLSPGRKGTLSCRASQSVSGGSLAWYQQKPGQAPRLLIYDSSTRATGVPGRFSGSGSETDFSLTISRLEAEDFAVYYCQQYGTSQSTFGQGTRLEIR
KC	V4K3	1219291	20.345	89.5	DIVLTQSPGRFSLSPEERATLSCRASQSVSGGYVAWYQQKAGQAPRLPIYDYVSRATGVPGRFSGSGSETDFSLTISRLEPEDFAVYYCQQYGTSQSTFGQGTRLEIR
KC	V6K1	1899647	9.655	82.1	EMVLTQSPGTLSLSPGEGATLSCRASQSVVYNSLAWYQQRPGQAPRLAILAASRRATGIPDRFSGSGSGTDFTLTISRLEPEDFAVYYCQQYGTSQGTFGQGTKVEIK
KC	V6K2	132411	3.103	85.5	EIVLTQSPGTLSLSPGERATLSCRASQSVSSSYLAWYQQKPGQAPRLLIYGAYIRATGIPDRFSGSGSGTDFTLTISRLEPEDFAVYYCQQYGTSQGTFGQGTKVEIK
KC	V6K3	2357651	10.345	81.5	RMVLTQSPGTLSLSPGEGATLSCGASQSVVYNSLAWYQQRPGQAPRLVILAASRRAAGIPDRFSGSGSGTDFTLTISRLEPEDFAVYYCQQYGTSQGTFGQGTKVEIK

Supplementary Table 5. Evaluation of neutralization breadth and potency of PGZL1, 4E10 and VRC01 on 130-member cross-clade pseudovirus panel using TZM-bl neutralization assay.

Key (IC_{50}/IC_{80} in μg/ml)

<0.1	0.1-1	1-10	10-50	50-200

		IC_{50} (μg/ml)					IC_{80} (μg/ml)				
Clade	Virus	PGZL1 _gVmDmJ	PGZL1	H4K3	4E10	VRC01	PGZL1 _gVmDmJ	PGZL1	H4K3	4E10	VRC01
A	NIH-065_191955 A11	33.5	1.89	0.476	0.011	0.091	119	8.56	2.23	0.157	0.467
A	NIH-063_0330.v4.c3	>200	22.1	2.63	0.963	0.033	>200	>50	10.5	4.17	0.130
A	NIH-066_191084 B7.19	>200	30.9	1.17	1.23	0.049	>200	>50	5.94	9.04	0.188
A	NAC_BG505	>200	16.7	2.40	1.15	0.056	>200	>50	9.75	4.88	0.213
A	NIH-057_MS208.A1	>200	10.1	0.726	0.678	0.063	>200	45.6	2.84	2.86	0.252
A	NIH-064_0260.v5.c1	>200	>50	7.75	0.227	0.124	>200	>50	35.3	6.08	0.516
A	NAC_KNH1144	>200	>50	18.0	11.5	0.134	>200	>50	>50	47.9	0.535
A	NAC_94UG103	>200	24.1	9.37	1.77	0.187	>200	>50	36.6	7.48	0.752
A	NAC_92RW020	>200	20.8	3.86	2.14	0.206	>200	>50	15.5	9.61	0.811
A1	NIH-060_Q769ENVd22	>200	20.2	3.19	1.61	0.025	>200	>50	12.9	6.57	0.092
A1	NIH-058_Q23ENV17	>200	16.5	3.13	1.29	0.038	>200	>50	12.7	5.37	0.148
A1	NIH-056_Q168ENVa2	>200	21.7	2.69	3.78	0.056	>200	>50	10.9	15.1	0.218
A1	NIH-061_Q259ENVd2.17	>200	>50	9.87	7.12	0.114	>200	>50	38.1	28.9	0.449
A1	NIH-059_Q461ENVe2	>200	20.2	3.59	2.33	0.195	>200	>50	14.3	9.31	0.776
A1U	NIH-117_T278.5	>200	23.5	3.49	1.48	>50	>200	>50	14.0	6.03	>50
AC	NIH-100_6041.v3.c23	>200	>50	2.66	5.48	0.023	>200	>50	10.9	22.2	0.087
AC	NIH-099_3301.v1.c24	>200	>50	19.8	20.3	0.112	>200	>50	>50	>50	0.463
AC	NIH-102_6545.v4.c1	>200	>50	7.56	2.35	>50	>200	>50	29.6	13.3	>50
AC	NIH-101_6540.v4.c1	>200	>50	5.50	5.63	>50	>200	>50	22.7	36.1	>50
ACD	NIH-103_0815.v3.c3	>200	18.7	5.17	1.20	0.019	>200	>50	20.5	6.31	0.070
ACD	NIH-104_3103.v3.c10	>200	>50	18.0	8.11	1.53	>200	>50	>50	32.4	6.21
AE	NIH-073_C3347.c11	10.2	0.055	0.036	0.034	0.063	41.3	0.263	0.165	0.137	0.254
AE	NIH-080_BJOX025000.01.1	14.6	7.13	0.379	0.377	6.04	59.9	29.3	1.53	1.59	24.8
AE	NIH-075_CNE8	18.2	0.247	0.041	0.087	0.126	72.2	0.945	0.210	0.270	0.514
AE	NAC_92TH021	20.9	2.12	0.235	0.348	0.324	79.9	8.27	0.926	1.38	1.31
AE	NIH-081_BJOX028000.10.3	49.4	5.16	0.583	0.450	0.141	199	20.9	2.38	1.88	0.573
AE	NIH-078_BJOX015000.11.5	79.3	2.59	0.079	0.165	0.060	>200	10.5	0.296	0.647	0.242
AE	NIH-079_BJOX010000.06.2	89.1	1.66	0.199	0.438	1.77	>200	6.60	0.794	1.73	7.79
AE	NIH-074_C4118.c09	106	8.37	0.424	0.322	0.050	>200	33.3	1.76	1.17	0.202
AE	NIH-069_C1080.c03	118	1.73	0.171	0.328	1.40	>200	6.93	0.690	1.36	5.70
AE	NIH-077_BJOX009000.02.4	161	3.97	0.522	0.389	0.565	>200	15.8	2.12	1.62	2.34
AE	NIH-068_620345.c01	170	12.6	1.18	0.663	>50	>200	>50	4.95	3.06	>50
AE	NIH-076_CNE5	>200	24.6	1.37	1.34	0.059	>200	>50	6.58	5.90	0.238
AE	NIH-070_R2184.c04	>200	12.9	0.976	0.223	0.103	>200	>50	5.15	0.762	0.413
AE	NIH-072_R3265.c06	>200	28.0	1.68	0.491	0.126	>200	>50	8.33	2.00	0.502
AE	NIH-071_R1166.c01	>200	4.87	0.623	0.383	0.488	>200	19.5	2.52	1.77	1.95
AG	NIH-115_928.28	32.4	1.38	0.252	0.112	0.249	125	5.45	1.01	0.438	0.990
AG	NIH-118_T255.34	38.7	3.35	0.453	0.326	0.229	148	13.2	1.78	1.38	0.925
AG	NIH-116_T263.8	107	2.02	0.166	0.206	0.124	>200	8.18	0.719	0.927	0.505
AG	NIH-108_T235.47	139	1.54	0.538	0.248	0.021	>200	6.62	2.21	1.07	0.084
AG	NIH-114_T257.31	>200	29.6	2.65	1.03	1.61	>200	>50	11.0	4.71	6.57
AG	NIH-107_T251.18	>200	43.0	4.52	0.905	2.02	>200	>50	17.9	3.97	8.63
AG	NIH-106_T250.4	>200	13.9	2.53	0.756	>50	>200	>50	10.4	3.42	>50
AG	NIH-119_T211.9	>200	13.2	2.66	1.20	12.8	>200	>50	10.5	5.08	>50
B	NAC_MN	1.21	0.023	0.005	0.005	0.010	19.3	0.564	0.135	0.135	0.196
B	NAC_HxB2	5.64	0.121	0.026	0.021	0.022	22.1	0.485	0.113	0.089	0.081
B	NIH-012_1012.11.TC21.3257	77.3	13.3	2.51	2.06	0.081	>200	>50	10.0	8.25	0.318
B	NAC_ADA	88.5	5.36	0.932	0.836	0.314	>200	21.8	3.71	3.34	1.31
B	NAC_WITO.33	112	3.56	2.59	0.435	0.055	>200	15.1	10.4	1.82	0.228
B	NIH-006_AC10.0.29	116	4.64	0.872	0.666	0.867	>200	18.8	3.50	2.73	3.52
B	NIH-003_SC422661.8	145	16.8	3.66	2.78	0.124	>200	>50	14.5	11.1	0.481
B	NAC_89.6	162	25.3	5.02	2.77	0.807	>200	>50	19.9	10.9	3.30
B	NAC_BaL.26	>200	>50	6.16	4.20	0.010	>200	>50	25.2	17.1	0.037
B	NAC_RHPA4.7	>200	>50	7.93	4.66	0.035	>200	>50	31.7	19.3	0.137
B	NIH-014_6244.13.B5.4567	>200	1.31	0.353	0.077	0.045	>200	5.19	1.51	0.373	0.193
B	NAC_TRJO.58	>200	>50	4.88	2.97	0.057	>200	>50	19.9	11.6	0.223
B	NAC_92BR020	>200	>50	10.3	2.57	0.059	>200	>50	39.7	13.5	0.230
B	NIH-008_WEAU.d15.410.787	>200	10.0	1.52	0.621	0.076	>200	39.6	6.30	2.59	0.306
B	NAC_REJO.67	>200	10.7	3.39	1.87	0.106	>200	40.2	13.8	8.62	0.414
B	NIH-009_1006.11.C3.1601	>200	24.6	5.69	5.66	0.145	>200	>50	23.0	22.7	0.571
B	NAC_SS1196.1	>200	5.40	0.640	0.990	0.166	>200	22.2	2.76	3.96	0.659
B	NAC_YU2	>200	>50	1.68	15.9	0.123	>200	>50	20.8	>50	0.690
B	NAC_JRCSF	>200	13.4	1.69	0.823	0.172	>200	>50	7.58	3.92	0.690
B	NAC_JRFL	>200	>50	12.6	17.8	0.141	>200	>50	>50	>50	0.741
B	NIH-004_PVO.4	>200	49.0	7.05	9.59	0.192	>200	>50	27.5	38.7	0.790
B	NAC_DH12	>200	4.71	1.54	1.17	0.227	>200	19.0	6.35	4.89	0.898
B	NIH-005_TRO.11	>200	2.88	0.669	0.769	0.231	>200	11.8	2.68	3.08	0.912
B	NAC_SF162	>200	12.8	2.43	1.97	0.262	>200	48.6	10.3	8.26	1.03
B	NIH-011_1056.10.TA11.1826	>200	3.72	0.595	0.688	0.372	>200	14.9	2.38	2.81	1.48
B	NIH-016_SC05.8C11.2344	>200	5.77	1.43	0.828	0.508	>200	24.6	5.71	3.44	1.98
B	NIH-013_6240.08.TA5.4622	>200	35.8	6.49	9.78	0.604	>200	>50	26.4	40.3	2.62
B	NIH-007_CAAN5342.A2	>200	34.0	7.61	2.84	0.691	>200	>50	30.6	12.1	2.79
B	NIH-010_1054.07.TC4.1499	>200	5.15	1.23	1.35	0.909	>200	20.2	4.93	5.43	3.62
B	NIH-001_6535.3	>200	1.42	0.652	0.519	0.946	>200	4.69	1.88	3.29	3.70
B	NIH-015_62357.14.D3.4589	>200	27.2	4.53	2.87	1.74	>200	>50	18.1	11.6	6.95
B	NIH-002_QHO692 .42	>200	42.0	4.54	6.19	1.96	>200	>50	18.1	25.0	7.93
BC	NIH-050_CNE21	97.8	4.23	1.02	0.513	0.212	>200	17.8	4.36	2.15	0.848
BC	NIH-054_CNE53	>200	6.00	0.842	0.380	0.042	>200	23.9	3.40	1.52	0.166
BC	NIH-055_CNE58	>200	3.55	0.975	0.563	0.083	>200	14.5	4.10	2.24	0.331
BC	NIH-048_CNE19	>200	3.12	1.07	0.310	0.127	>200	12.1	4.30	1.43	0.512
BC	NIH-053_CNE52	>200	24.3	4.16	0.954	0.142	>200	>50	16.7	3.85	0.565
BC	NIH-051_CNE17	>200	12.4	1.87	0.526	0.219	>200	48.6	7.46	2.23	0.904

TABLE S5 (CONT'D)

Key (IC_{50}/IC_{80} in μg/ml)

<0.1	0.1-1	1-10	10-50	50-200

Clade	Virus	IC_{50} (μg/ml)					IC_{80} (μg/ml)				
		PGZL1 _gVmDmJ	PGZL1	H4K3	4E10	VRC01	PGZL1 _gVmDmJ	PGZL1	H4K3	4E10	VRC01
BC	NIH-052_CNE30	>200	40.5	3.49	4.17	0.593	>200	>50	14.1	16.6	2.38
BC	NIH-049_CNE20	>200	2.42	0.799	0.383	4.85	>200	9.50	3.26	1.58	20.2
C	NIH-030_HIV-0013095.2.11	0.552	0.008	0.070	0.001	0.0002	4.60	0.113	0.305	0.258	0.058
C	NIH-026_ZM135M.PL10a	0.879	0.296	0.026	0.133	0.102	3.13	2.08	0.195	0.936	0.379
C	NIH-017_Du156.12	6.97	0.623	0.270	0.101	0.059	27.7	2.50	1.08	0.447	0.230
C	NIH-019_Du422.1	56.8	7.51	1.41	1.42	>50	>200	30.5	5.65	5.66	>50
C	NIH-018_Du172.17	75.2	0.424	0.106	0.102	>50	151	1.60	0.422	0.474	>50
C	NIH-032_HIV-16845.2.22	93.2	0.4244	0.1047	0.097	0.9682	>200	2.468	0.718	0.653	4.252
C	NIH-039_Ce2060 G9	140	15.0	0.541	1.24	0.109	>200	>50	2.48	5.10	0.450
C	NAC_93IN905	159	0.404	0.361	0.110	0.070	>200	2.20	1.51	0.472	0.280
C	NIH-029_HIV-001428.2.42	>200	>50	9.12	5.54	0.019	>200	>50	35.9	24.7	0.068
C	NIH-041_BF1266.431a	>200	48.8	5.59	2.47	0.028	>200	>50	22.3	10.0	0.110
C	NIH-023_ZM249M.PL1	>200	12.7	2.27	1.44	0.050	>200	>50	8.97	5.77	0.197
C	NIH-031_HIV-16055.2.3	>200	10.9	1.38	1.59	0.053	>200	42.6	5.68	6.57	0.206
C	NIH-043_ZM249M.B10	>200	6.26	1.47	1.11	0.071	>200	25.6	5.88	4.55	0.275
C	NIH-025_ZM109F.PB4	>200	7.36	1.54	0.703	0.074	>200	29.7	6.50	3.17	0.296
C	NIH-040_Ce703010054 2A2	>200	48.9	7.78	2.05	0.139	>200	>50	30.1	12.5	0.595
C	NIH-036_Ce2010 F5	>200	>50	5.20	14.4	0.153	>200	>50	20.9	>50	0.625
C	NIH-020_ZM197M.PB7	>200	1.91	0.805	0.226	0.170	>200	7.66	3.27	1.08	0.682
C	NIH-044_ZM247F.F7	>200	12.0	3.49	1.85	0.236	>200	48.2	14.0	7.50	0.945
C	NIH-046_1394C9G1(Rev.)	>200	7.87	2.67	2.58	0.280	>200	34.3	10.8	10.4	1.12
C	NIH-047_Ce704809221 1B3	>200	0.738	0.440	0.134	0.320	>200	3.10	1.81	0.557	1.28
C	NAC_IAVIC22	>200	15.2	2.20	1.21	0.348	>200	>50	9.00	6.11	1.37
C	NIH-034_Ce0393 C3	>200	13.5	1.73	0.606	0.370	>200	>50	7.68	2.56	1.46
C	NIH-045_7030102001E5(Rev.)	>200	49.6	4.63	3.41	0.380	>200	>50	18.4	15.7	1.49
C	NIH-021_ZM214M.PL15	>200	28.7	6.52	5.31	0.612	>200	>50	26.3	21.2	2.42
C	NIH-024_ZM53M.PB12	>200	>50	12.7	10.4	0.917	>200	>50	>50	42.6	3.63
C	NIH-035_Ce1176 A3	>200	31.7	11.3	1.74	1.25	>200	>50	44.2	7.36	4.99
C	NIH-022_ZM233M.PB6	>200	5.90	1.75	1.05	1.41	>200	23.6	7.36	4.99	5.69
C	NIH-027_CAP45.G3	>200	18.3	1.95	0.886	1.92	>200	>50	8.34	4.19	10.1
C	NIH-042_ZM246F.D5	>200	19.9	3.13	1.68	10.7	>200	>50	12.7	6.70	43.7
C	NHI-028_CAP210.E8	>200	16.7	1.82	0.847	>50	>200	>50	7.22	3.53	>50
C	NIH-038_Ce1172 H1	>200	0.948	0.351	8.81	>50	>200	6.60	1.34	4.56	>50
CD	NIH-095_6480.v4.c25	>200	15.0	0.335	0.561	0.014	>200	>50	1.61	3.04	0.054
CD	NIH-096_6952.v1.c20	>200	0.890	0.195	0.721	0.025	>200	4.70	0.779	3.50	0.097
CD	NIH-097_6811.v7.c18	>200	>50	9.54	2.74	0.140	>200	>50	39.2	17.0	0.558
CD	NIH-094_3817.v2.c59	>200	20.1	0.597	0.417	>50	>200	>50	2.74	2.12	>50
CD	NIH-098_89.F1 2 25	>200	>50	8.60	5.11	>50	>200	>50	33.2	21.7	>50
D	NIH-089_3016.v5.c45	74.8	2.40	0.375	0.915	0.058	>200	10.6	1.59	3.73	0.223
D	NIH-090_A07412M1.vrc12	>200	13.9	1.01	2.58	0.051	>200	>50	4.05	10.4	0.216
D	NIH-091_231965.c01	>200	>50	6.70	13.8	0.183	>200	>50	27.0	>50	0.743
G	NIH-086_X2131 C1 B5	14.3	0.885	0.148	0.194	0.073	57.7	3.50	0.626	0.765	0.284
G	NIH-082_X1193 c1	29.7	5.31	0.632	0.359	0.098	121	21.3	2.62	1.43	0.408
G	NIH-084_X1254 c3	>200	45.5	6.06	2.88	0.039	>200	>50	24.2	11.8	0.149
G	NIH-083_P0402 c2 11	>200	8.29	0.382	0.084	0.040	>200	32.3	1.82	0.363	0.164
G	NIH-087_P1981 C5 3	>200	1.39	0.147	0.099	0.203	>200	5.55	0.581	0.406	0.800
G	NIH-088_X1632 S2 B10	>200	15.5	3.02	2.57	0.338	>200	>50	11.9	10.4	1.35
G	NIH-085_X2088 c9	>200	>50	19.3	>50	>50	>200	>50	>50	>50	>50
-	NAC_AMLV	>200	>50	>50	>50	>50	>200	>50	>50	>50	>50
-	NAC_VSV	>200	>50	>50	>50	>50	>200	>50	>50	>50	>50

IC_{50}/IC_{80} and Percent of Viruses Neutralized

	PGZL1 _gVmDmJ	PGZL1	H4K3	4E10	VRC01	PGZL1 _gVmDmJ	PGZL1	H4K3	4E10	VRC01
# Viruses	130	130	130	130	130	130	130	130	130	130
**Total Viruses Neutralized**										
IC_{50}/IC_{80} < 200 μg/ml	36	ND	ND	ND	ND	16	ND	ND	ND	ND
IC_{50}/IC_{80} < 50 μg/ml	15	109	130	129	118	6	61	124	124	117
IC_{50}/IC_{80} < 10 μg/ml	5	54	122	122	116	2	26	72	92	113
IC_{50}/IC_{80} < 1 μg/ml	2	13	48	64	104	0	5	16	20	81
IC_{50}/IC_{80} < 0.1 μg/ml	0	3	7	10	44	0	0	0	1	10
**Percent of Viruses Neutralized**										
IC_{50}/IC_{80} < 200 μg/ml	28	ND	ND	ND	ND	12	ND	ND	ND	ND
IC_{50}/IC_{80} < 50 μg/ml	12	84	100	99	91	5	47	95	95	90
IC_{50}/IC_{80} < 10 μg/ml	4	42	94	94	89	2	20	55	71	87
IC_{50}/IC_{80} < 1 μg/ml	2	10	37	49	80	0	4	12	15	62
IC_{50}/IC_{80} < 0.1 μg/ml	0	2	5	8	34	0	0	0	1	8
Median IC_{50}/IC_{80}	76.3	9.2	1.69	1.01	0.139	66.0	13.2	6.58	4.17	0.561
IC_{50}/IC_{80} (Geometric Mean)	40.2	6.06	1.39	0.911	0.160	49.0	9.59	5.35	3.71	0.654

Supplementary Table 6. Impact of antibody washout on neutralization of HIV by PGZL1 gVmDmJ germline revertant

Isolates	IC_{50} (µg/ml)		Fold change[a]
	no wash	wash	
T255	63.1	>200	>3.2
X2131	85.7	>200	>2.3
BJOX028000	37.1	>200	>5.4
928.28	0.50	36.5	73
1193	33.2	196	5.9
Du156.12	6.47	103	16
BJOX025000	6.99	63	9
HxB2	2.12	11.9	5.6
92Th021	22.3	74.6	3.4
Du156.12- (VRC01)	0.11	0.16	1.5

[a]MPER accessibility was determined by washing antibody-virion mixture prior to infecting TZM-bl cells. Pseudoviruses were incubated with antibody at 37°C for 30 min, and antibody-virion mixture was washed or not washed prior to infecting target cells. Impact of antibody wash-out on virus neutralization is shown as the fold change in IC_{50}: (IC_{50} wash)/(IC_{50} no wash).

Supplementary Table 7. X-ray data collection and refinement statistics for PGZL1 Fab variants

**Complex**	**PGZL1 Fab**	**PGZL1 Fab - $MPER_{671-683}$**	**PGZL1 Fab - $MPER_{671-683}$ - 06:0 PA**	**H4K3 Fab**	**H4K3 Fab - $MPER_{671-683}$**	**H4K3 Fab - 06:0 PA**	**PGZL1_gVmDmJ Fab - Protein G**	**PGZL1_gVmDmJ Fab - $MPER_{671-683}$ - 06:0 PA**
**Lipid**	**-**	**-**	**06:0 PA**	**-**	**-**	**06:0 PA**	**-**	**06:0 PA**
**Reservoir condition**[*]	20% PEG 4000, 20% glycerol, 0.08 M sodium acetate pH 4.6, 0.16 M ammonium sulfate	20 % PEG 4000, 20% glycerol, 0.08 M sodium acetate pH 4.6, 0.16 M ammonium sulfate	0.1 M sodium acetate pH 5.5, 12% 2-methyl-2,4-pentanediol	30% PEG 2000 MME, 0.1 M sodium acetate pH 4.6, 0.2 M ammonium sulfate	0.2 M ammonium dihydrogen phosphate, 22% PEG3350	1.44 M ammonium sulfate, 0.01M cobalt chloride, 0.1 M MES pH 5.83	20% PEG3350, 0.2 M lithium citrate	0.1 M sodium acetate pH 4.6, 0.2 M ammonium sulfate, 30% PEG 2000 monomethyl ether
**Cryoprotectant**	26% glycerol	26% glycerol	20% ethylene glycol	26% glycerol	26%glycerol	26% glycerol	26% glycerol	26%glycerol
**Data collection**								
X-ray source beamline	SSRL 9-2	SSRL 9-2	SSRL 9-2	SSRL 12-2	SSRL 12-2	SSRL 12-2	SSRL 12-2	SSRL 12-2
Detector	Dectris PILATUS 6M	Dectris PILATUS 6M	Dectris PILATUS 6M	Dectris PILATUS 6M	Dectris PILATUS 6M	Dectris PILATUS 6M	Dectris PILATUS 6M	Dectris PILATUS 6M
Wavelength (Å)	0.97946	0.97946	0.97946	0.97946	0.97946	0.97946	0.97946	0.97946
Temperature (K)	110	110	110	110	110	110	110	110
No. of crystals	1	1	1	1	2	1	1	1
Space group	C2	$P3_121$	C2	$P2_1$	P1	$I2_13$	$P2_1$	C2
Unit cell (*a*, *b*, *c*; Å) (α, β, γ; °)	87.59, 96.02, 67.32 90, 120.18, 90	113.53, 113.53, 300.54 90, 90, 120	181.86, 43.93, 154.53 90, 92.49, 90	52.36, 64.98, 68.00 90, 105.81, 90	55.34, 58.03, 98.73 87.94, 76.09, 72.52	276.20, 276.20, 276.20 90, 90, 90	78.84, 107.49, 131.96 90, 94.61, 90	208.36, 37.92, 133.52 90, 93.68, 90
Resolution range (Å)	37.04-1.40	44.13 -3.65	44.10-3.42	35.51-1.45	36.15-1.98	39.06-3.11	39.67-2.47	39.77-2.60
Highest resolution shell[a]	1.42-1.40	3.71-3.65	3.48-3.42	1.48-1.45	2.01-1.98	3.19-3.11	2.53-2.47	2.67-2.60
No. measurements	298,709 (10,546)	124,715 (5,414)	69,732 (3,564)	207,268 (4,944)	276,332 (9,263)	201,006 (13,965)	268,463 (18,466)	90,892 (6,566)
No. unique reflections	89,730 (3,906)	24,133 (1,203)	15,913 (810)	74,084 (2,908)	72,569 (2,807)	61,847 (4,503)	77,485 (5,460)	30,972 (2,317)
Redundancy	3.3 (2.7)	5.2 (4.5)	4.4 (4.4)	2.8 (1.7)	3.8 (3.3)	3.3 (3.1)	3.5 (3.4)	2.9 (2.8)
Completeness (%)	95.4 (83.2)	93.1 (95.2)	93.4 (95.3)	95.4 (75.4)	91.3 (70.7)	98.2 (97.8)	97.7 (94.0)	93.8 (95.2)
R_{sym}[b]	8.2 (89.1)	18.9 (92.7)	21.0 (88.7)	5.6 (69.9)	8.3 (23.0)	11.4 (120.5)	9.9 (94.7)	15.3 (98.4)
R_{pim}[c]	5.2 (60.9)	9.1 (43.4)	11.0 (46.6)	3.9 (61.5)	4.6 (14.1)	7.3 (79.2)	6.2 (59.7)	10.7 (73.1)
$CC_{1/2}$[d]	95.5 (80.7)	87.8 (68.5)	87.5 (57.2)	86.5 (49.5)	94.9 (97.7)	99.5 (42.5)	99.5 (52.6)	98.7 (57.1)
Signal to noise ($\langle I/\sigma I \rangle$)	7.8 (1.4)	7.3 (1.8)	6.6 (1.8)	10.1 (1.1)	12.6 (6.0)	9.8 (1.0)	9.2 (1.5)	6.4 (1.2)
Solvent content (%)	51.1	56.4	60.9	47.1	58.0	73.0	64.4	54.2
**Refinement**								
No. reflections used for refinement	89,715	24,115	15,906	74,047	72,539	61,813	77,455	30,948
R_{crys}[e] (%)	14.8	26.0	21.4	15.6	17.2	18.6	16.9	25.7
R_{free}[f] (%)	18.2	28.9	26.4	19.1	21.1	22.8	21.9	31.0
**Model composition (asymmetric unit)**								
Fab or Fab-peptide complex	1	4	2	1	2	4	3	2
Protein G	n/a	n/a	n/a	n/a	n/a	n/a	3	n/a
Waters	579	-	-	472	480	34	396	71
Lipid	n/a	n/a	2	n/a	n/a	8	n/a	-
SO4 or PO4	1 (SO_4)	4 (SO_4)	n/a	2 (SO_4)	6 (PO_4)	14 (SO_4)	n/a	4 (SO_4)
**B-values**								
Wilson plot ($Å^2$)	17	95	79	17	27	90	50	43
Overall mean isotropic ($Å^2$)	24	98	79	24	33	95	52	49
Fab / Protein G mean isotropic ($Å^2$)	22 / (n/a)	99 / (n/a)	74 / (n/a)	24 / (n/a)	33 / (n/a)	94 / (n/a)	51 / 56	48 / (n/a)
Peptide mean isotropic ($Å^2$)	n/a	78	97	n/a	42	n/a	n/a	71
Ions mean isotropic ($Å^2$)	46	104	n/a	75	60	166	n/a	51
06:0 PA(s) mean isotropic ($Å^2$)	n/a	n/a	173	n/a	n/a	150	n/a	-
Waters mean isotropic ($Å^2$)	37	-	n/a	37	37	61	49	38
**Estimated coordinate error r.m.s.d. from ideal values (Å)**								
Bond lengths (Å)	0.011	0.003	0.002	0.003	0.008	0.002	0.005	0.002
Bond angles (°)	1.12	0.88	0.53	0.93	0.92	0.53	0.82	0.54
**Ramachandran**								
Most favored regions (%)	96.1	94.7	95.3	96.7	97.1	95.3	95.9	94.5
Additional allowed regions (%)	3.3	4.0	3.3	2.7	1.7	3.6	3.3	4.2
Disallowed regions (%)	0.6	1.3	1.4	0.6	1.2	1.1	0.8	1.3

[a] Values in parentheses correspond to the highest resolution shells

[b] $R_{sym} = \Sigma_{hkl} \Sigma_{j=1,N} |\langle I_{hkl} \rangle - I_{hklj}| / \Sigma_{hkl} \Sigma_{j=1,N} |I_{hklj}|$, where the outer sum (hkl) is taken over the unique reflections

[c] $R_{pim} = \Sigma_{hkl} [1/(N-1)]^{1/2} \Sigma_{i=1,N} |I_{hkli} - \langle I_{hkl} \rangle| / \Sigma_{hkl} \Sigma_{i=1,N} |I_{hkli}|$

[d] $CC_{1/2}$ = Pearson Correlation Coefficient between two random half datasets

[e] $R_{cryst} = \Sigma_{hkl} ||F_{o,hkl}| - k|F_{c,hkl}|| / \Sigma_{hkl} |F_{o,hkl}|$, where $|F_{o,hkl}|$ and $|F_{c,hkl}|$ are the observed and calculated structure factor amplitudes, respectively

[f] R_{free} , as for R_{cryst}, but for a set of reflections (5% of the total) omitted from refinement

[*] Drop conditions prepared as described in Experimental Procedures

Supplementary Table 8. Primers used in this study.

Oligo Name	Oligo Sequence (5'-3')	
5' L-VH 1	ACAGGTGCCCACTCCCAGGTGCAG	
5' L-VH 3	AAGGTGTCCAGTGTGARGTGCAG	
5' L-VH 4/6	CCCAGATGGGTCCTGTCCCAGGTGCAG	
5' L-VH 5	CAAGGAGTCTGTTCCGAGGTGCAG	
3' CH1	GGAAGGTGTGCACGCCGCTGGTC	
5' LVL 1	GGTCCTGGGCCCAGTCTGTGCTG	
5' LVL 2	GGTCCTGGGCCCAGTCTGCCCTG	
5' LVL 3	GCTCTGTGACCTCCTATGAGCTG	
5' LVL 4/5	GGTCTCTCTCSCAGCYTGTGCTG	PCR1
5' LVL 6	GTTCTTGGGCCAATTTTATGCTG	
5' LVL 7	GGTCCAATTCYCAGGCTGTGGTG	
5' LVL 8	GAGTGGATTCTCAGACTGTGGTG	
3' CL	CACCAGTGTGGCCTTGTTGGCTTG	
5' LVK 1/2	ATGAGGSTCCCYGCTCAGCTGCTGG	
5' LVK 3	CTCTTCCTCCTGCTACTCTGGCTCCCAG	
5' LVK 4	ATTTCTCTGTTGCTCTGGATCTCTG	
3' CK 543	GTTTCTCGTAGTCTGCTTTGCTCA	
5'AgeI VH1/5	CTGCAACCGGTGTACATTCCGAGGTGCAGCTGGTGCAG	
5'AgeI VH3	CTGCAACCGGTGTACATTCTGAGGTGCAGCTGGTGGAG	
5'AgeI VH4	CTGCAACCGGTGTACATTCCCAGGTGCAGCTGCAGGAG	
5'AgeI VH3-23	CTGCAACCGGTGTACATTCTGAGGTGCAGCTGTTGGAG	
5'AgeI VH4-34	CTGCAACCGGTGTACATTCCCAGGTGCAGCTACAGCAGTG	
3' IgG (internal)	GTTCGGGGAAGTAGTCCTTGAC	
5' AgeI VL 1	CTGCTACCGGTTCCTGGGCCCAGTCTGTGCTGACKCAG	
5' AgeI VL 2	CTGCTACCGGTTCCTGGGCCCAGTCTGCCCTGACTCAG	PCR2
5' AgeI VL 3	CTGCTACCGGTTCTGTGACCTCCTATGAGCTGACWCAG	
5' AgeI VL 4/5	CTGCTACCGGTTCTCTCTCSCAGCYTGTGCTGACTCA	
5' AgeI VL 6	CTGCTACCGGTTCTTGGGCCAATTTTATGCTGACTCAG	
5' AgeI VL 7/8	CTGCTACCGGTTCCAATTCYCAGRCTGTGGTGACYCAG	
3' XhoI CL	CTCCTCACTCGAGGGYGGGAACAGAGTG	
5' Pan VK	ATGACCCAGWCTCCABYCWCCCTG	
3' CK 494	GTGCTGTCCTTGCTGTCCTGCT	
SL 5′ VH1	gggttttccttgttgctattctcgagggtgtccagtgtCAGGTGCAGCTGGTGCAG	
SL 5′ VH1/5	gggttttccttgttgctattctcgagggtgtccagtgtGAGGTGCAGCTGGTGCAG	
SL 5′ VH3	gggttttccttgttgctattctcgagggtgtccagtgtGAGGTGCAGCTGGTGGAG	
SL 5′ VH3–23	gggttttccttgttgctattctcgagggtgtccagtgtGAGGTGCAGCTGTTGGAG	
SL 5′ VH4	gggttttccttgttgctattctcgagggtgtccagtgtCAGGTGCAGCTGCAGGAG	
SL 5′ VH 4–34	gggttttccttgttgctattctcgagggtgtccagtgtCAGGTGCAGCTACAGCAGTG	
SL 5′ VH 1–18	gggttttccttgttgctattctcgagggtgtccagtgtCAGGTTCAGCTGGTGCAG	
SL 5′ VH 1–24	gggttttccttgttgctattctcgagggtgtccagtgtCAGGTCCAGCTGGTACAG	
SL 5′ VH3–33	gggttttccttgttgctattctcgagggtgtccagtgtCAGGTGCAGCTGGTGGAG	
SL 5′ VH 3–9	gggttttccttgttgctattctcgagggtgtccagtgtGAAGTGCAGCTGGTGGAG	
SL 5′ VH4–39	gggttttccttgttgctattctcgagggtgtccagtgtCAGCTGCAGCTGCAGGAG	
SL 5′ VH 6–1	gggttttccttgttgctattctcgagggtgtccagtgtCAGGTACAGCTGCAGCAG	
SL 5' VH7-4-1	gggttttccttgttgctattctcgagggtgtccagtgtCAGGTGCAGCTGGTGCAATC	
SL 3′ JH 1/2/4/5	GATGGGCCCTTGGTGCTAGCTGAGGAGACGGTGACCAG	
SL 3′ JH 3	GATGGGCCCTTGGTGCTAGCTGAAGAGACGGTGACCATTG	
SL 3′ JH 6	GATGGGCCCTTGGTGCTAGCTGAGGAGACGGTGACCGTG	
SL 5′ VL 1	TTTTTCTAGTAGCAACTGCAACCGGTGTACACCAGTCTGTGCTGACTCAG	
SL 5′ VL 2	TTTTTCTAGTAGCAACTGCAACCGGTGTACACCAGTCTGCCCTGACTCAG	
SL 5′ VL 3	TTTTTCTAGTAGCAACTGCAACCGGTGTACACTCCTATGAGCTGACTCAG	PCR 3
SL 5′ VL 4/5	TTTTTCTAGTAGCAACTGCAACCGGTGTACACCAGCCTGTGCTGACTCA	
SL 5′ VL 6	TTTTTCTAGTAGCAACTGCAACCGGTGTACACAATTTTATGCTGACTCAG	
SL 5′ VL 7/8	TTTTTCTAGTAGCAACTGCAACCGGTGTACACCAGACTGTGGTGACTCAG	
SL 3′ CL	TGTTGGCTTGAAGCTCCTCACTCGAGGGCGGGAACAGAGTG	
SL 5′ VK 1–5	TTTTTCTAGTAGCAACTGCAACCGGTGTACACGACATCCAGATGACCCAGTC	
SL 5′ VK 1–9	TTTTTCTAGTAGCAACTGCAACCGGTGTACACGACATCCAGTTGACCCAGTCT	
SL 5′ VK 1D–43	TTTTTCTAGTAGCAACTGCAACCGGTGTACACGCCATCCGGATGACCCAGTC	
SL 5′ VK 2–24	TTTTTCTAGTAGCAACTGCAACCGGTGTACACGATATTGTGATGACCCAGAC	
SL 5′ VK 2–28	TTTTTCTAGTAGCAACTGCAACCGGTGTACACGATATTGTGATGACTCAGTC	
SL 5′ VK 2–30	TTTTTCTAGTAGCAACTGCAACCGGTGTACACGATGTTGTGATGACTCAGTC	
SL 5′ VK 3–11	TTTTTCTAGTAGCAACTGCAACCGGTGTACACGAAATTGTGTTGACACAGTC	
SL 5′ VK 3–15	TTTTTCTAGTAGCAACTGCAACCGGTGTACACGAAATAGTGATGACGCAGTC	
SL 5′ VK 3–20	TTTTTCTAGTAGCAACTGCAACCGGTGTACACGAAATTGTGTTGACGCAGTCT	
SL 5′ VK 4–1	TTTTTCTAGTAGCAACTGCAACCGGTGTACACGACATCGTGATGACCCAGTC	
SL 3′ JK 1/4	AAGACAGATGGTGCAGCCACCGTACGTTTGATCTCCACCTTGGTC	
SL 3′ JK 2	AAGACAGATGGTGCAGCCACCGTACGTTTGATCTCCAGCTTGGTC	
SL 3′ JK 3	AAGACAGATGGTGCAGCCACCGTACGTTTGATATCCACTTTGGTC	
SL 3′ JK 5	AAGACAGATGGTGCAGCCACCGTACGTTTAATCTCCAGTCGTGTC	
