# Supplementary material for: An MPER antibody neutralizes HIV-1 using germline features shared among donors
Source: Nat Commun. 2019 Nov 26;10:5389. doi: 10.1038/s41467-019-12973-1 (PMC6879610; doi:10.1038/s41467-019-12973-1)
Supplement: Supplementary file 3 — Reporting Summary [file 41467_2019_12973_MOESM3_ESM.pdf]

nature research

Corresponding author(s): Jiang Zhu
Ian A. Wilson
Michael B. Zwick

Last updated by author(s): Oct 8, 2019

Reporting Summary

Nature Research wishes to improve the reproducibility of the work that we publish. This form provides structure for consistency and transparency in reporting. For further information on Nature Research policies, see Authors & Referees and the Editorial Policy Checklist.

Statistics

For all statistical analyses, confirm that the following items are present in the figure legend, table legend, main text, or Methods section.

n/a	Confirmed	
☐	☒	The exact sample size (n) for each experimental group/condition, given as a discrete number and unit of measurement
☐	☒	A statement on whether measurements were taken from distinct samples or whether the same sample was measured repeatedly
☐	☒	The statistical test(s) used AND whether they are one- or two-sided<br>*Only common tests should be described solely by name; describe more complex techniques in the Methods section.*
☒	☐	A description of all covariates tested
☒	☐	A description of any assumptions or corrections, such as tests of normality and adjustment for multiple comparisons
☒	☐	A full description of the statistical parameters including central tendency (e.g. means) or other basic estimates (e.g. regression coefficient) AND variation (e.g. standard deviation) or associated estimates of uncertainty (e.g. confidence intervals)
☒	☐	For null hypothesis testing, the test statistic (e.g. F, t, r) with confidence intervals, effect sizes, degrees of freedom and P value noted<br>*Give P values as exact values whenever suitable.*
☒	☐	For Bayesian analysis, information on the choice of priors and Markov chain Monte Carlo settings
☒	☐	For hierarchical and complex designs, identification of the appropriate level for tests and full reporting of outcomes
☒	☐	Estimates of effect sizes (e.g. Cohen's d, Pearson's r), indicating how they were calculated

Our web collection on statistics for biologists contains articles on many of the points above.

Software and code

Policy information about availability of computer code

Data collection

X-ray diffraction data were collected at Stanford Synchrotron Radiation Lightsource, SLAC National Accelerator Laboratory on SSRL 12-2 and SSRL 9-2 beam-lines. The diffraction data sets were auto-indexed, integrated and scaled using the program HKL2000 (http://www.hkl-xray.com/hkl-2000) or XDS (http://xds.mpimfheidelberg.mpg.de/).

Cryo-EM micrographs were collected on a Titan Krios transmission electron microscope (Thermo Fisher Scientific) and K2 Summit direct electron detector (Gatan) using Leginon automated image acquisition software (http://emg.nysbc.org/redmine/projects/leginon/wiki/Leginon_Homepage).

Next-generation sequencing was performed on a Ion GeneStudio S5 Next-Generation Sequencing System.

Flow cytometry: cells were acquired and analyzed by using NovoCyte (ACEA Biosciences).

Data analysis

X-ray diffraction structure where solved by molecular replacement using Phaser (http://www.phaser.cimr.cam.ac.uk/index.php/Phaser_Crystallographic_Software). The structures were refined using the program PHENIX (https://www.phenix-online.org/), model building was done in COOT (https://www2.mrclmb.cam.ac.uk/personal/pemsley/coot/) and structural figures were generated using PyMol (https://pymol.org/2/).

Cryo-EM frames were aligned and dose weighted with MotionCor2 (http://msg.ucsf.edu/em/software/motioncor2.html) and CTF models for each micrograph were calculated using GCTF (https://www.mrclmb.cam.ac.uk/kzhang/). Particles were picked using DoGPicker (https://omictools.com/dog-pickertool). The initial round of 2D classification was done with CryoSPARC (https://cryosparc.com/) and the 3D processing with Relion (https://www2.mrclmb.cam.ac.uk/relion/index.php?title=Download_%26_install#Getting_started).

Next-generation sequencing data was processed, annotated, and analyzed using Antibodyomics pipeline (He et al., 2017).

Flow cytometry data were analyzed using FlowJo software (Tree Star) (http://docs.flowjo.com/vx/faq/general-faq/tree-star-flowjo/).

Lipid insertion propensity scores were calculated using the MPEx (Membrane Protein Explorer) software (http://blanco.biomol.uci.edu/mpex/)

CCS sequences were constructed using the PacBio SMRTportal software (version 2.3) (https://www.pacb.com/). The Robust Amplicon Denoising algorithm (https://github.com/MurrellGroup/RobustAmpliconDenoising.jl) was used for error correction and MAFFT (https://mafft.cbrc.jp/alignment/software/), was used to construct a multiple sequence alignment. Phylogenies were reconstructed using FastTree v2.1 (http://www.microbesonline.org/fasttree/), and visualized with FigTree (http://tree.bio.ed.ac.uk/software/figtree/). Geno2Pheno 2.5 (https://coreceptor.geno2pheno.org/) was used to predict co-receptor tropism. Env sequences, and browser-based visualizations, are available at http://flea.murrell.group/view/PG13/sequences.

Prism6 (GraphPad) was used to calculate EC50/IC50 values on the ELISA/Neutralization experiments.

CHARMM (https://www.charmm.org) was used to construct the model of the PGZL1.H4K3 interaction with HIV MPER epitope on membrane.

ForteBio Data Analysis 7 software was used for analysis of data in bio-layer interferometry experiments.

Image Lab software (Bio-Rad) was used to analyze the western blots imaged using a Chemidoc XRS (Bio-Rad).

IMGT online tools (www.imgt.org) were used to analyze heavy chain and light chain variable regions.

DNASTAR software was used to analyze sequences

For manuscripts utilizing custom algorithms or software that are central to the research but not yet described in published literature, software must be made available to editors/reviewers. We strongly encourage code deposition in a community repository (e.g. GitHub). See the Nature Research guidelines for submitting code & software for further information.

Data

Policy information about availability of data

All manuscripts must include a data availability statement. This statement should provide the following information, where applicable:

- Accession codes, unique identifiers, or web links for publicly available datasets
- A list of figures that have associated raw data
- A description of any restrictions on data availability

The PGZL1 and its variants HCs and LCs variable region sequences have been deposited into Genbank, accession MK497833-MK497838.
The atomic coordinates and structure factors of PGZL1 variants have been deposited in the Protein Data Bank, with accession codes: 6O3D (PGZL1); 6O3G (PGZL1 - MPER671-683); 6O3J (PGZL1 - MPER671-683 - 06:0 PA); 6O3K (H4K3); 6O3L (H4K3 - MPER671-683); 6O3U (H4K3 - 06:0 PA); 6O41 (PGZL1 gVmDmJ - Protein G); 6O42 (PGZL1 gVmDmJ - MPER671-683 – 06:0 PA).
The Cryo-EM reconstruction of full-length AMC011-PGT151-PGZL1 complex has been deposited to Electron Microscopy Data Bank with accession code EMD-0620.
Datasets generated during and/or analysed during the current study are included in a supplemental Source Data file.

Field-specific reporting

Please select the one below that is the best fit for your research. If you are not sure, read the appropriate sections before making your selection.

☒ Life sciences ☐ Behavioural & social sciences ☐ Ecological, evolutionary & environmental sciences

For a reference copy of the document with all sections, see nature.com/documents/nr-reporting-summary-flat.pdf

Life sciences study design

All studies must disclose on these points even when the disclosure is negative.

Sample size	Figure 3D, 7 groups at 7 samples per group<br>Figure S3D 4 groups at 89 samples per group
Data exclusions	no
Replication	Figure 3D involves "Area under the curve (AUC)" so each value (sample) is the result of 6 points on a curve. Multiple non-specific antigens are also included in the experiment, providing multiple independent measurements of non-specific binding.
Randomization	no
Blinding	no

Reporting for specific materials, systems and methods

We require information from authors about some types of materials, experimental systems and methods used in many studies. Here, indicate whether each material, system or method listed is relevant to your study. If you are not sure if a list item applies to your research, read the appropriate section before selecting a response.

Materials & experimental systems

n/a	Involved in the study
☐	☒ Antibodies
☐	☒ Eukaryotic cell lines
☒	☐ Palaeontology
☒	☐ Animals and other organisms
☐	☒ Human research participants
☒	☐ Clinical data

Methods

n/a	Involved in the study
☒	☐ ChIP-seq
☐	☒ Flow cytometry
☒	☐ MRI-based neuroimaging

Antibodies

Antibodies used	Monoclonal anti-HIV-1 Env 4E10<br>Monoclonal anti-HIV-1 Env VRC01<br>Monoclonal anti-HIV-1 Env 10E8<br>Monoclonal anti-HIV-1 Env PGT145<br>Monoclonal anti-HIV-1 Env b6<br>Monoclonal anti-HIV-1 Env 4E10_gVgDgJ<br>Monoclonal anti-HIV-1 Env 4E10_gVmDmJ<br>Monoclonal anti-HIV-1 Env 4E10_L100cF<br>Monoclonal anti-HIV-1 Env 10E8_gVmDmJ<br>Monoclonal anti-HIV-1 Env PGZL1<br>Monoclonal anti-HIV-1 Env H4K3<br>Monoclonal anti-HIV-1 Env PGZL1_gVmDmJ<br>Monoclonal anti-HIV-1 Env PGZL1_gVgDmJ<br>Monoclonal anti-HIV-1 Env PGZL1_gVgDgJ<br>Monoclonal anti-HIV-1 Env<br>FITC conjugated AffiniPure Goat Anti-Human IgG, F(ab')2 fragment specific (Jackson ImmunoResearch Cat# 109-096-008);<br>HRP- conjugated AffiniPure Goat Anti-Human IgG, Fc fragment specific (Jackson ImmunoResearch Cat# 109-035-098);
Validation	Validation of the antibodies that were purchased from commercial sources (Jackson ImmunoResearch) were described by the vendor. Monoclonal HIV antibodies from other researchers were either obtained by original laboratories, NIH ARRRP, or produced in-house and titered side-by-side with the former version(s) in ELISA and neutralization assays to ensure batch-to-batch consistency.

Eukaryotic cell lines

Policy information about cell lines

Cell line source(s)	Human: HEK293T (ATCC Cat# CRL-3216, RRID: CVCL_0063);<br>Human: HeLa-derived TZM-bl (NIH AIDS Reagent Program Cat# 8129-442, RRID: CVCL_B478);<br>Human: FreeStyle 293F (Thermo Fisher Cat#R79007; RRID: CVCL_D603);<br>Human: Expi293F (Life Technologies Cat# A14527)<br>Human: V4 cell (Stano, 2017 Journal of Virology)<br>Human: MPER-TM cell (this study)
Authentication	Tzm-bl cells and 293T cells, and other cell lines were prepared sterilely in multiple aliquots from validated sources (NIH ARRRP, Thermo Fisher) and then stored under liquid nitrogen. Cell lines were passaged at the recommended frequency and seeding density, in fresh media, and were routinely inspected under a microscope in the lab; cells were discarded when the recommended passage number was reached.
Mycoplasma contamination	Cell lines were tested and found to be negative for mycoplasma contamination.
Commonly misidentified lines<br>(See ICLAC register)	n/a

Human research participants

Policy information about studies involving human research participants

Population characteristics	IAVI Protocol G project;<br>More than 1,800 healthy, HIV-positive volunteers. A paper describing the IAVI Protocol G Cohort has been published: Simek et al., J Virol 2009, 83:7337-7348.
Recruitment	volunteers who are HIV infected

Ethics oversight	Donor PG13 was from IAVI-sponsored Protocol G cohort in South Africa. Blood samples were collected with written, informed consent, and the study was reviewed and approved by the relevant Ethics and Research Committees, as described above in the paper by Simek et al, 2009.

Note that full information on the approval of the study protocol must also be provided in the manuscript.

Flow Cytometry

Plots

Confirm that:

☒ The axis labels state the marker and fluorochrome used (e.g. CD4-FITC).

☒ The axis scales are clearly visible. Include numbers along axes only for bottom left plot of group (a 'group' is an analysis of identical markers).

☒ All plots are contour plots with outliers or pseudocolor plots.

☒ A numerical value for number of cells or percentage (with statistics) is provided.

Methodology

Sample preparation	Datasets generated during and/or analysed during the current study are available from the corresponding author on reasonable request.<br><br>Sorting experiment: Cells were stained using the LIVE/DEAD Fixable Near-IR Dead Cell Kit (Life Technologies) for 30 min on ice according to the manufacturer’s instructions. Cells were then labeled with antibodies to surface markers along with MPER probes for 1 h in Brilliant Staining buffer (BD Biosciences) on ice.<br><br>Analyzing experiment: A total of 10-7 cells of stable cell lines expressing HIV-1 MPER-TM or Comb-mut Env (V4) were washed in PBS and labeled with Fixable Aqua Dead Cell Stain (Life Technologies) for the exclusion of dead cells. Cells were washed in FACS buffer (PBS supplemented with 2% heat-inactivated FBS) and stained with monoclonal antibody. After another wash, cells were stained using APC-conjugated goat anti-human Fc. Soluble CD4 was incubated with cells for 30 min prior to staining cells with antibody.
Instrument	Sorting experiment: BD FACSAria III sorter<br>Analyzing experiment: NovoCyte (ACEA Biosciences)
Software	FlowJo software (Tree Star)
Cell population abundance	*Describe the abundance of the relevant cell populations within post-sort fractions, providing details on the purity of the samples and how it was determined.*
Gating strategy	Sorting experiment: CD19+/CD20+, CD3-/CD8-, CD14-/CD16-, IgG+, IgD-/IgM-; MPER tetramer-BV421, MPER tetramer-APC (double positive)<br>Analyzing experiment: Amcyan-/APC+

☒ Tick this box to confirm that a figure exemplifying the gating strategy is provided in the Supplementary Information.
